# Chloronitramide Anion Quantitation in Tap Waters by Ion Chromatography with Electrical Conductivity and Ultraviolet Absorbance Detection

**DOI:** 10.1021/acs.estlett.5c01218

**Published:** 2026-01-19

**Authors:** Jason A. Thornhill, Juliana R. Laszakovits, Barrett E. Johnson, Justin R. Chimka, Julian L. Fairey

**Affiliations:** † Department of Civil Engineering, 3341 University of Arkansas, Fayetteville, Arkansas 72701, United States; ‡ Institute of Biogeochemistry and Pollutant Dynamics, ETH Zurich, 8092 Zurich, Switzerland; § Department of Industrial Engineering, University of Arkansas, Fayetteville, Arkansas 72701, United States

**Keywords:** Drinking Water, Disinfection Byproducts, Chloramine Decomposition, Disinfectant Chemistry, Analytical Methods

## Abstract

Chloronitramide anion (Cl–N–NO_2_^–^) is a recently discovered inorganic chloramine decomposition product, with prior quantitation by hydrophilic interaction liquid chromatography–ultrahigh-resolution mass spectrometry (HILIC–UHRMS). Here, Cl–N–NO_2_^–^ quantitation was evaluated by ion chromatography (IC) separation with electrical conductivity (EC) and ultraviolet absorbance at 243 nm (UV_243_) detection. Using a 5 mL injection loop and a 14.4 mM Na_2_CO_3_ eluent, the Cl–N–NO_2_^–^ method detection limit was 9.3 μg L^–1^ by IC-EC and 6.2 μg L^–1^ by IC-UV_243_. Matrix testing with Cl–N–NO_2_^–^ spiked at 15, 25, 50, and 100 μg L^–1^ into 12 waters in triplicate matched the expected Cl–N–NO_2_^–^ for IC-EC (R^2^ = 0.994) and IC-UV_243_ (R^2^ = 0.993). Cl–N–NO_2_^–^ was found by HILIC–UHRMS in all tested tap waters disinfected with chloramines (n = 8) at 22–314 μg L^–1^ and free chlorine (n = 3) at 2–7 μg L^–1^, and strongly correlated with IC-EC (R^2^ = 0.991; average residual, $\overset{®}{r_{i}\;}$ = +3.2 μg L^–1^) and IC-UV_243_ (R^2^ = 0.997; $\overset{®}{r_{i}\;}$ = 0.0 μg L^–1^). This is the first report of Cl–N–NO_2_^–^ in free chlorine systems and concentrations ≳200 μg L^–1^ in chloramine systems. These IC methods enable widescale collection of Cl–N–NO_2_^–^ occurrence data, with IC-UV_243_ subject to lesser measurement bias.

## Graphical abstract

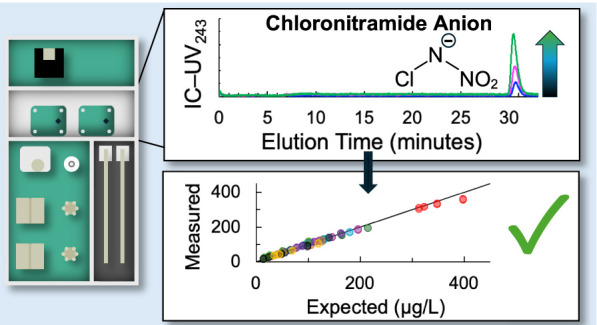

## 1 Introduction

After ∼45 years since it was first detected in chloraminated water^1^ and more than 30 years since its prior characterization efforts,^2–5^ chloronitramide anion (Cl–N–NO_2_^–^) was identified as a decomposition product of inorganic chloramines.^6^ In a limited survey of chloraminated tap waters in the United States, Cl–N–NO_2_^–^ was found in all samples tested (n = 40) at a median concentration of 23 μg L^–1^ and first and third quartiles of 1.3 and 92 μg L^–1^, respectively. Although its toxicity is currently unknown, Cl–N–NO_2_^–^ forms at levels comparable to some regulated disinfection byproducts (DBPs) but lower than others. For example, the Environmental Protection Agency (EPA) maximum contaminant levels (MCLs) for the mass-based sums of four trihalomethanes is 80 μg L^–1^ and five haloacetic acids is 60 μg L^–1^;^7^ however, the chlorite MCL is over 1 order of magnitude greater, at 1 mg L^–1^. The Cl–N–NO_2_^–^ formation pathway contrasts that of most other DBPs, which are formed by reactions between a disinfectant and source water precursors, such as natural organic matter (NOM) and/or bromide.^8,9^ Cl–N–NO_2_^–^ is formed by monochloramine (NH_2_Cl) or dichloramine (NHCl_2_) reacting with nitronium cation (NO_2_^+^),^6^ a reactive nitrogen species formed during chloramine decomposition.^10,11^ In this regard, Cl–N–NO_2_^–^ is an intrinsic DBP,^12^ like chlorate and perchlorate in hypochlorite feedstocks.^13–16^ As chloramines are inherently unstable and autodecompose under typical conditions in drinking water systems,^17–19^ Cl–N–NO_2_^–^ is likely present in all waters where chloramines are used. This includes systems where chloramines are formed intentionally and free chlorine systems containing source water ammonia.^20^ To facilitate Cl–N–NO_2_^–^ occurrence studies and better understand its formation in tap water, analytical methods are needed for Cl–N–NO_2_^–^ quantitation that leverage common instrumentation in drinking water laboratories.

The discovery of Cl–N–NO_2_^–^ was supported by its quantitation in chloraminated tap waters by hydrophilic interaction liquid chromatography–ultrahigh-resolution mass spectrometry (HILIC–UHRMS).^6^ This method has a limit of detection (LOD) of 0.17 μg L^–1^ and a limit of quantitation (LOQ) of 0.58 μg L^–1^. However, the instrumentation required for HILIC–UHRMS (i.e., orbitrap mass spectrometers) is not widely accessible due to its high capital cost and operation and maintenance requirements, limiting collection of Cl–N–NO_2_^–^ occurrence data. Cl–N–NO_2_^–^ reference materials were previously isolated from mixtures containing common anions (e.g., chloride, nitrite, nitrate, sulfate, phosphate) at concentrations up to ∼100 mM using ion chromatography (IC) with electrical conductivity (EC) and ultraviolet absorbance (UV) detectors.^6^ Given Cl–N–NO_2_^–^ is an anion and has a molar absorptivity (ε) peak maxima at 243 nm of ε_243_ = 5,310 M^–1^ cm^–1^,^6^ Cl–N–NO_2_^–^ quantitation may be possible by IC-EC and/or IC-UV_243_, but the applicability of these methods at μg L^–1^ levels is unknown. IC methods could be a cost-effective alternative to MS-based methods and adopted at drinking water utilities and commercial water analysis laboratories, enabling widescale Cl–N–NO_2_^–^ occurrence studies. The objective of this study is to assess IC-EC and IC-UV_243_ for Cl–N–NO_2_^–^ quantitation in tap waters at μg L^–1^ levels. Cl–N–NO_2_^–^ quantitation by IC-EC and IC-UV_243_ was compared to HILIC–UHRMS across an unprecedented range of Cl–N–NO_2_^–^ concentrations in tap waters from U.S. residential homes.

## 2 Materials and Methods

Lab-grade water was obtained from a Thermo Scientific Smart2Pure 6 UV water purification system with a resistivity of 18.2 MΩ cm and was used for all aqueous phase preparations. This section's remainder describes the (1) IC methods for (a) Cl–N–NO_2_^–^ reference material isolation from the lab-generated mixtures and (b) Cl–N–NO_2_^–^ quantitation at μg L^–1^ levels, and (2) waters used for Cl–N–NO_2_^–^ reproducibility and matrix testing and comparisons of the IC methods to HILIC-UHRMS.

### 2.1 Ion Chromatography System

A Metrohm 850 Professional IC equipped with an autosampler, suppressor, two electrical conductivity detectors, and an 887 Professional UV/vis detector was used to isolate Cl–N–NO_2_^–^ standard reference materials and quantify Cl–N–NO_2_^–^ in tap waters. Two Metrosep A Supp 7 separation columns were used, each 250 mm in length with a 4.0 mm diameter, one for Cl–N–NO_2_^–^ isolation that had been used extensively (>10,000 injections) and regenerated to maintain adequate anion separation and the second newly acquired for Cl–N–NO_2_^–^ quantitation in drinking water matrices. Both IC columns consist of a PEEK housing packed with a poly­(vinyl alcohol) carrier material with quaternary ammonium groups, 5 μm particle size, 15 MPa maximum pressure, 1.0 mL min^–1^ maximum flow rate, pH range 3–12, and temperature range of 20–60 °C. Na_2_CO_3_ eluent concentrate (Metrohm) was diluted 100× to 3.6 mM for Cl–N–NO_2_^–^ isolation and 25× to 14.4 mM for Cl–N–NO_2_^–^ quantitation.

### 2.2 Cl–N–NO_2_^–^ Standard Reference Material Isolation by Ion Chromatography

Cl–N–NO_2_^–^ was formed at ∼14.5–20.2 mM by reacting 254 mM NH_2_Cl with equimolar NO_2_^–^ as detailed in prior work^6^ in 1 M carbonate buffer at pH 8.5 instead of 1 M phosphate buffer at pH 7.2. Carbonate buffer was used because it was partially removed by the IC suppression system and is compatible with ongoing bioassays. ^15^N-labeled Cl–N–NO_2_^–^ was used as an internal standard for HILIC-UHRMS quantitation, prepared by reacting 254 mM ^15^NH_2_Cl with equimolar NO_2_^–^ in 1 M phosphate at pH 7.2.^6^ The IC parameters for isolation are detailed in the Supporting Information, S1.1.2, Figures s1–s4, and Table s1 and include a 100 μL injection loop, 10 mL sample volume, 50 °C column temperature, 60 min run time, 0.7 mL min^–1^ eluent flow rate, and 3.6 mM Na_2_CO_3_ eluent strength. Following IC separation and peak collection (see Figure s4), the Cl–N–NO_2_^–^ isolate was standardized by measuring the absorbance at 243 nm in a 1 cm quartz cuvette with a Shimadzu UV-2450 spectrophotometer and quantified using the previously determined molar absorptivity of 5,310 M^–1^ cm^–1^.^6^

### 2.3 Cl–N–NO_2_^–^ Quantitation by Ion Chromatography

Cl–N–NO_2_^–^ quantitation by IC-EC and IC-UV_243_ peak area and height used the same IC system as used for isolation equipped with a newly acquired Metrosep A Supp 7 separation column. The Supporting Information, S1.1.3 and Table s1 details the IC parameters used for Cl–N–NO_2_^–^ quantitation which included a 5 mL injection loop, 10 mL sample volume, 45 °C column temperature, 35 min run time, 0.7 mL min^–1^ eluent flow rate, and 14.4 mM Na_2_CO_3_ eluent strength. S1.2 details the preparation of Cl–N–NO_2_^–^ standard curves and S1.3 details the method detection limit (MDL),^21^ LOD, and LOQ^22^ determinations.

### 2.4 Sample Waters for Cl–N–NO_2_^–^ Reproducibility and Matrix Testing

Lab-grade water amended with 1 mM borate buffer at pH 9 was used for the reproducibility testing with the Cl–N–NO_2_^–^ standards at 15, 25, 50, and 100 μg L^–1^. Triplicates of each standard were run on seven different days to assess the IC-EC and IC-UV_243_ signal drift.

One synthetic groundwater (GW), eight chloraminated tap waters, and three chlorinated tap waters were used for Cl–N–NO_2_^–^ matrix spikes (i.e., repeatability testing)^23^ with the Cl–N–NO_2_^–^ standards spiked at 15, 25, 50, and 100 μg L^–1^ in triplicate. Tap waters were anonymized by their two-letter state abbreviation and numbered to distinguish multiple samples from a given state and included AR, TX-1, PA, TX-2, GA-1, GA-2, SC, CA, TX-3, OK, and MN. Additional details regarding tap water collection are provided in the Supporting Information, S1.4. Synthetic groundwater was prepared from ASTM D1141–98 ^24^ sea-salt (Lake Products Company LLC, Florissant, MO) dissolved in lab-grade water at a 100X dilution factor and amended with nitrate at ∼1 mg L^–1^ as N following others.^25^ The tap waters were characterized for pH, total chlorine by DPD,^26^ NH_2_Cl by indophenol,^27^ and Cl–N–NO_2_^–^ by IC and HILIC-UHRMS. The Supporting Information details the reproducibility and matrix testing (S1.5) and the HILIC–UHRMS method (S1.6).

## 3 Results and Discussion

### 3.1 Cl–N–NO_2_^–^ MDL, LOD, and LOQ

We separated Cl–N–NO_2_^–^ from common anions by IC with detection at μg L^–1^ levels by EC and UV_243_. The elution time for Cl–N–NO_2_^–^ was ∼30 min (Figure s5), eluting ∼ 15 min after the last of the common anions. In each IC run, Cl–N–NO_2_^–^ standards eluted at similar times (∼30 min, Figure s6), with similar full-width at half maximums (∼0.7 min) for IC-EC and IC-UV_243_. Figures s7–s15 show 17 Cl–N–NO_2_^–^ standard curves (5–500 μg L^–1^) measured by IC-EC and IC-UV_243_ between October and November 2025. The standard curve R^2^ values ranged from 0.990–1.000 by IC-EC and 0.995–1.000 by IC-UV_243_. Table s2 shows the IC-based MDL, LOD, and LOQ determinations for the two detectors (EC and UV_243_) and two quantitation methods (peak area and peak height). The peak area results are discussed herein while the peak height results are in the Supporting Information. For IC-EC, the MDL (9.3 μg L^–1^), LOD (7.4 μg L^–1^), and LOQ (8.2 μg L^–1^) were like those for IC-UV_243_, with an MDL (6.2 μg L^–1^), LOD (5.8 μg L^–1^), and LOQ (8.3 μg L^–1^). The lowest of these LOD and LOQ values (5.8 and 8.2 μg L^–1^) are greater than those previously reported for HILIC–UHRMS (LOD = 0.17 μg L^–1^ and LOQ = 0.58 μg L^–1^),^6^ but are nevertheless suitable for Cl–N–NO_2_ quantification in most chloraminated tap waters (≳ 10 μg L^–1^).^6^

### 3.2 Reproducibility Tests

Figure s16 shows Cl–N–NO_2_^–^ standards at 15, 25, 50, and 100 μg L^–1^ in 1 mM borate buffer at pH 9 analyzed in triplicate on seven different days to assess the reproducibility of the IC-EC and IC-UV_243_ methods. The average residual, $\overset{®}{r_{i}\;}$ , was calculated as the total sum of the residuals divided by the number of observations for each standard (n = 21) and is shown parenthetically above each data set in Figure s16 to illustrate the presence of a bias, with $\overset{®}{r_{i}\;}$ values further from zero indicating a greater bias. For Cl–N–NO_2_^–^ quantitation by peak area, $\overset{®}{r_{i}\;}$ for the four Cl–N–NO_2_^–^ standards had a lower bias for IC-UV_243_ (i.e., +0.3, –0.6, –1.2, and –0.4 μg L^–1^, Figure s15C) than for IC-EC (i.e., +2.6, +0.7, –2.1, and –3.3 μg L^–1^, Figure s15A). Figure s15 indicates IC-UV_243_ has the lesser bias and hence the greatest reproducibility for Cl–N–NO_2_^–^ quantitation with $\overset{®}{r_{i}\;}$ of 0.3–1.2 μg L^–1^ in error from their target concentrations of 15–100 μg L^–1^.

### 3.3 Matrix Spike Tests

To assess matrix effects in the IC methods, Cl–N–NO_2_^–^ was added at concentrations of 15, 25, 50, and 100 μg L^–1^ into the synthetic GW, eight chloraminated tap waters, and three free chlorine tap waters. Table s3 shows their pH (7.3–8.8), total chlorine (≤3.0 mg L^–1^ as Cl_2_), monochloramine (≤2.7 mg L^–1^ as Cl_2_), and free chlorine (≤2.4 mg L^–1^ as Cl_2_). Figures s17–s28 show the IC-EC and IC-UV_243_ chromatograms of each tap water, the tap water spiked with 25 μg L^–1^ Cl–N–NO_2_^–^, and a 10 mg L^–1^ common anion standard. These chromatograms show Cl–N–NO_2_^–^ elution occurred after the last of the common anions by ∼15 min and certain tap waters (i.e., PA [Figure s19], GA-1 [Figure s21], SC [Figure s23], and MN [Figure s28]) contained unidentified peaks in the IC-EC profiles only that eluted after nitrate but did not coelute with Cl–N–NO_2_^–^. Figure 1 shows the IC-EC (Figure 1A) and IC-UV_243_ (Figure 1C) measured Cl–N–NO_2_^–^ vs the expected Cl–N–NO_2_^–^ and their corresponding residuals (Figures 1B and 1D). Figure s29 shows these data for peak height. The IC methods were used for the background Cl–N–NO_2_^–^ for the eight chloraminated tap waters with Cl–N–NO_2_^–^ > 20 μg L^–1^, and the HILIC–UHRMS determined background concentrations were used for the three free chlorine tap waters with Cl–N–NO_2_^–^ at 1.9–6.9 μg L^–1^. The measured Cl–N–NO_2_^–^ concentrations in the spiked waters ranged from ∼15–400 μg L^–1^ (n = 156). For Cl–N–NO_2_^–^ quantitation by IC-EC, the R^2^ of 0.994 (Figure 1A) indicates a strong correlation between the measured and expected Cl–N–NO_2_^–^ concentrations. Although the residuals had a negative bias across the entire range of expected Cl–N–NO_2_^–^ concentrations, the P value for the expected Cl–N–NO_2_^–^ concentrations <50 μg L^–1^ (P _< 50 μg L^–1^_) of 0.77 (Figure 1B) indicates IC-EC was unbiased in this lower concentration range. Likewise, for Cl–N–NO_2_^–^ quantitation by IC-UV_243_, Figure 1C shows a strong correlation coefficient (R^2^ = 0.993) and Figure 1D indicates unbiased residuals (P_<50 μg L^–1^_ = 0.14) for concentrations <50 μg L^–1^. Figure 1 indicates Cl–N–NO_2_^–^ quantitation by IC-EC and IC-UV_243_ had high precision and accuracy across a wide range of Cl–N–NO_2_^–^ (15–400 μg L^–1^) and unbiased residuals for concentrations <50 μg L^–1^.

**Figure 1:**
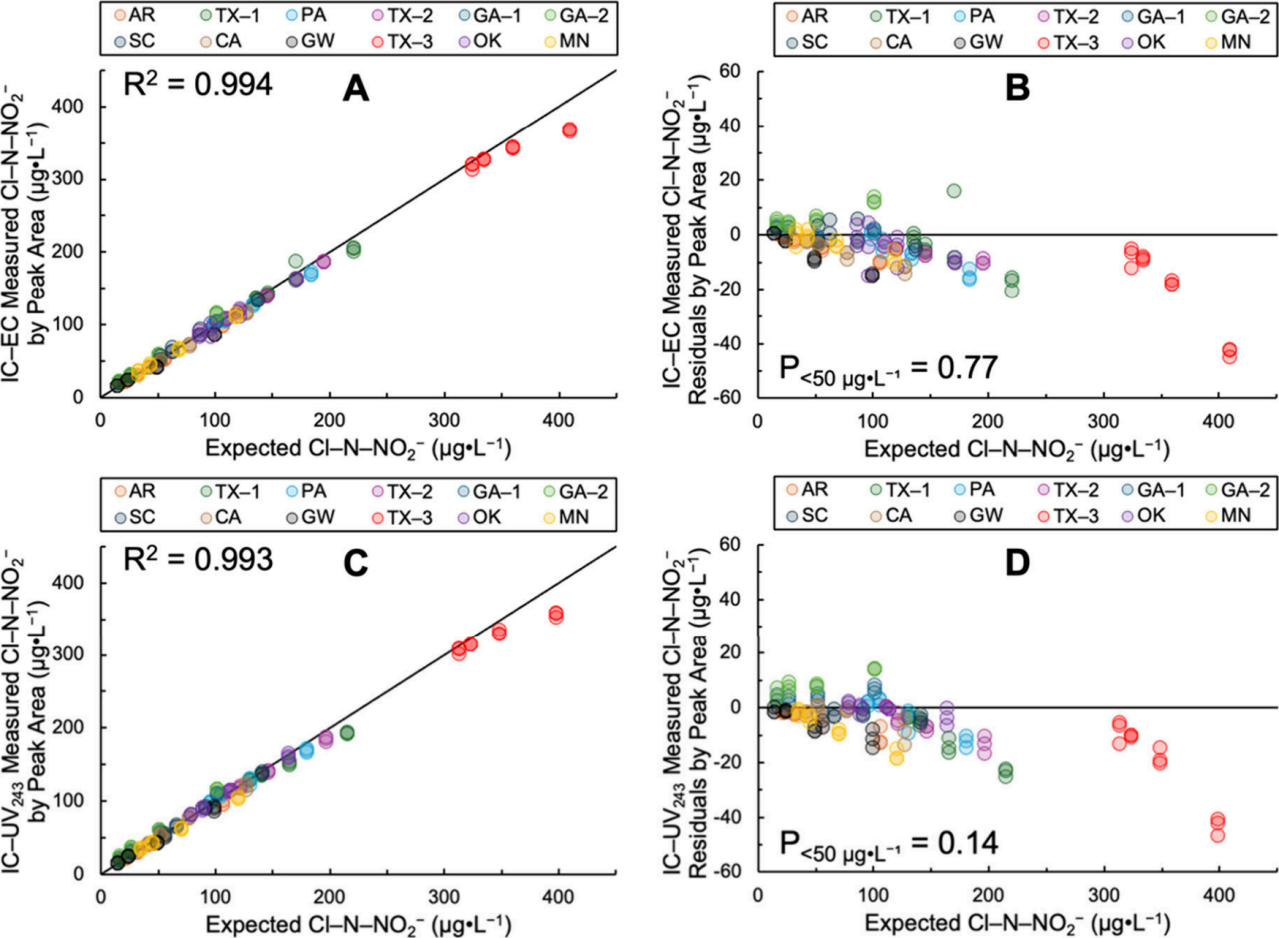
Ion chromatography-electrical conductivity (IC-EC, Panels A and B) and -ultraviolet absorbance at 243 nm (IC-UV_243_, Panels C and D) matrix testing with Cl–N–NO_2_^–^ spiked at 15, 25, 50, and 100 μg L^–1^ into 12 waters which included eight chloraminated tap waters (TX-1, PA, TX-2, SC, CA, TX-3, OK, and MN), three chlorinated tap waters (AR, GA-1, and GA-2), and one synthetic groundwater (GW) with no added disinfectant. (A) IC-EC measured by peak area vs the expected Cl–N–NO_2_^–^, (B) IC-EC residuals by peak area, (C) IC-UV_243_ measured by peak area vs the expected Cl–N–NO_2_^–^, and (D) IC-UV_243_ residuals by peak area. The solid black line in Panels A and C is the 1:1 line and shown with the correlation coefficient, R^2^. The P value at the α = 0.05 significance level is shown for expected Cl–N–NO_2_^–^ concentrations <50 μg L^–1^ (P_<50 μg L^–1^_) with a threshold value >0.05 indicating the residuals have a median indistinguishable from zero by the sign test^28^ and are therefore unbiased in that concentration range.

### 3.4 Tap Water Comparison of IC and HILIC–UHRMS

Figure 2 shows Cl–N–NO_2_^–^ concentrations in the 12 tap waters measured by IC-EC and IC-UV_243_ peak area compared to the HILIC–UHRMS method developed in prior work.^6^ Figure s30 shows these data for peak height.

**Figure 2:**
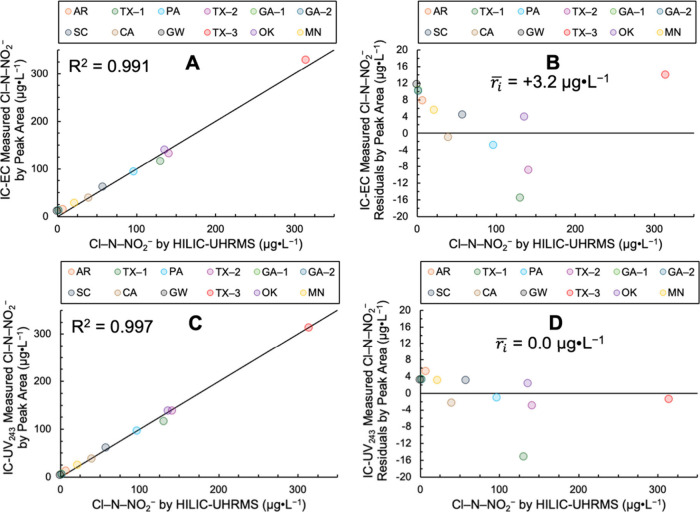
Ion chromatography-electrical conductivity (IC-EC, Panels A and B) and -ultraviolet absorbance at 243 nm (IC-UV_243_, Panels C and D) comparison testing with HILIC–UHRMS for Cl–N–NO_2_^–^ quantitation in 12 waters which included eight chloraminated tap waters (TX-1, PA, TX-2, SC, CA, TX-3, OK, and MN), three chlorinated tap waters (AR, GA-1, and GA-2), and one synthetic groundwater (GW) with no added disinfectant. (A) IC-EC measured by peak area vs Cl–N–NO_2_^–^ determined by HILIC–UHRMS, (B) IC-EC residuals by peak area, (C) IC-UV_243_ measured by peak area vs Cl–N–NO_2_^–^ determined by HILIC–UHRMS, and (D) IC-UV_243_ residuals by peak area. The solid black line in Panels A and C is the 1:1 line and shown with the correlation coefficient, R^2^. The average residual ( $\overset{®}{r_{i}\;}$ ; *n* = 12) in μg L^–1^ is shown in Panels B and D as a measure of the linear regression model bias and accuracy with values closer to zero indicating lesser overall bias.

Cl–N–NO_2_^–^ quantified using the IC methods tracked the 1:1 line across the range of Cl–N–NO_2_^–^ concentrations in the tap waters from not detected to 315 μg L^–1^, indicating they were strongly correlated with the HILIC–UHRMS measurements. For IC-EC, the R^2^ (n = 12) was 0.991 (Figure 2A) and the average residual ( $\overset{®}{r_{i}\;}$ ) was +3.2 μg L^–1^, indicating an overall positive bias. For IC-UV_243_, the R^2^ (n = 12) was 0.997 and the $\overset{®}{r_{i}\;}$ was 0.0 μg L^–1^, indicating no overall bias. Therefore, the accuracy and precision of Cl–N–NO_2_^–^ quantitation by IC-UV_243_ exceeded that of IC-EC for the tap waters tested. This is the first report, to the best of our knowledge, of Cl–N–NO_2_^–^ presence in chlorinated tap waters. Although the Cl–N–NO_2_^–^ concentrations are lower compared to chloraminated tap waters, this finding suggests that Cl–N–NO_2_^–^ exposure is more widespread and additional formation pathways such as breakpoint chlorination could be relevant, as previously speculated.^6^ The Cl–N–NO_2_^–^ concentration maximum observed in the chloraminated tap waters (315 μg L^–1^) is greater by a factor of about three compared to that in prior work,^6^ illustrating a pressing need for additional occurrence studies to capture its full range in water systems.

We demonstrated that IC-EC and IC-UV_243_ can be used to accurately quantify Cl–N–NO_2_^–^ in tap waters with minor modifications to the IC system (i.e., 5 mL injection loop and 14.4 mM Na_2_CO_3_ eluent) using standards formulated in 1 mM borate buffer at pH 9. While the lowest LOD (5.8 μg L^–1^) and LOQ (8.2 μg L^–1^) are greater than those for HILIC-UHRMS (0.17 and 0.58 μg L^–1^, respectively), the IC methods are accurate within an environmentally relevant range, with Cl–N–NO_2_^–^ at levels up to about 315 μg L^–1^. Spike recovery experiments indicate matrix effects were negligible for Cl–N–NO_2_^–^ quantitation by IC-EC and IC-UV_243_ for concentrations <50 μg L^–1^ (i.e., P_<50 μg L^–1^_ > 0.05). IC-based quantitation methods were strongly correlated with HILIC–UHRMS (R^2^ ≥ 0.991) and unbiased for quantitation by IC-UV_243_ peak area. Cl–N–NO_2_^–^ may continue to form in tap waters containing a chloramine residual, particularly at pH ≲ 8 where chloramines decompose more readily.^29^ A quenching agent/procedure has not yet been identified for chloramines that does not impact Cl–N–NO_2_^–^. Thus, the time between sample collection and analysis should be minimized (<1 day, if possible) to get the most accurate assessment of Cl–N–NO_2_^–^ occurrence until if/when a suitable sample preservation procedure is developed. Nevertheless, the IC methods developed and validated here can be adopted by water utilities and testing laboratories for widespread Cl–N–NO_2_^–^ monitoring campaigns.

## Supplementary Material



## Notes

Ion chromatography methods for Cl–N–NO_2_^–^ isolation and quantitation, standard curve preparation, MDL, LOD, and LOQ determinations, water sample collection, reproducibility, and matrix testing (PDF)
