# Supplementary material for: Chloronitramide Anion Quantitation in Tap Waters by Ion Chromatography with Electrical Conductivity and Ultraviolet Absorbance Detection
Source: Environ Sci Technol Lett. 2026 Jan 19;13(2):275–80. doi: 10.1021/acs.estlett.5c01218 (PMC12895537; doi:10.1021/acs.estlett.5c01218)
Supplement: Supplementary file 1 [file ez5c01218_si_001.pdf]

SUPPORTING INFORMATION:

Chloronitramide Anion Quantitation in Tap Waters by Ion Chromatography with Electrical Conductivity and Ultraviolet Absorbance Detection

AUTHORS:

Jason A. Thornhill, Juliana R. Laszakovits, Barrett E. Johnson, Justin R. Chimka, and Julian L. Fairey

CONTENTS

The Supporting Information (SI) contains the (1) materials and methods detailing the (i) IC methods for chloronitramide anion ($Cl\text{-}N\text{-}NO_2^-$) isolation and quantitation, (ii) $Cl\text{-}N\text{-}NO_2^-$ standard curve preparation, (iii) $Cl\text{-}N\text{-}NO_2^-$ method detection limit (MDL), limit of detection (LOD), and limit of quantitation (LOQ) calculations, (iv) water sample collection, and (v) $Cl\text{-}N\text{-}NO_2^-$ reproducibility and matrix testing; (2) results and discussion detailing $Cl\text{-}N\text{-}NO_2^-$ MDL, LOD, and LOQ and $Cl\text{-}N\text{-}NO_2^-$ reproducibility and matrix testing; (3) three tables; (4) thirty figures; and (5) references cited in the SI.

S1. Materials and Methods

S1.1 Chloronitramide Anion Isolation and Quantitation by Ion Chromatography

S1.1.1 Overview. One IC separation column was used for Cl-N-NO_2^- isolation and had been previously used extensively (> 10,000 injections) and regenerated as needed to maintain adequate anion separation; a second IC separation column was acquired new for Cl-N-NO_2^- quantitation in typical drinking water matrices and samples formulated with lab-grade water. MagIC Net 4.1 software was used for operating the IC and collecting and processing the data.

S1.1.2 Isolation Method. The IC method used in our prior work[1] was adopted here for isolation of the Cl-N-NO_2^- reference material from high concentrations of chloride, nitrite, nitrate and other common anions in the lab-generated mixtures. Cl-N-NO_2^- isolation was initially attempted with a 1 mL injection loop. Figure s1 shows the impact of eluent flowrate on Cl-N-NO_2^- separation, which was first assessed at the default flowrate of 0.7 mL•min^{-1}.

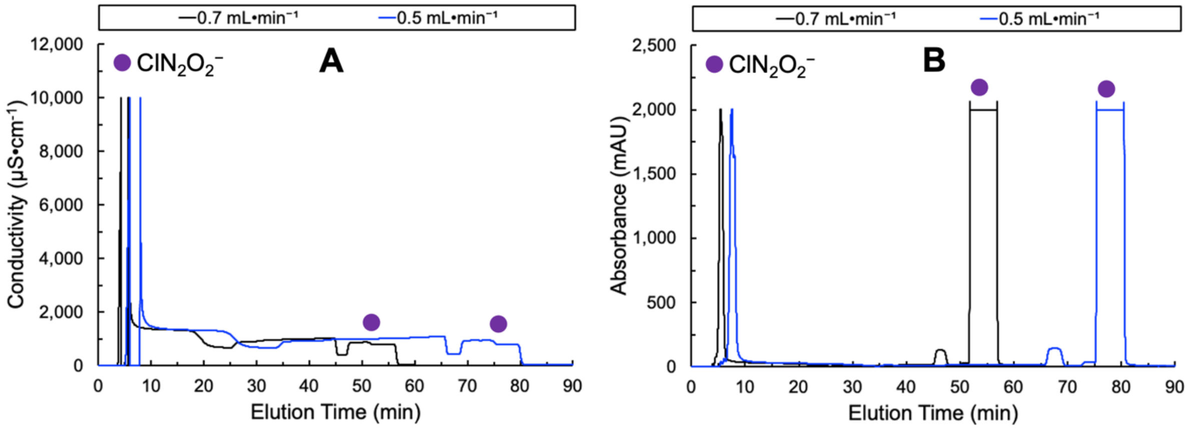

Figure s1. Ion chromatography (IC) method development for Cl-N-NO_2^- isolation from common anions showing the impact of eluent flowrate on Cl-N-NO_2^- elution ~50–58 minutes at 0.7 mL•min^{-1} and ~68–81 minutes at 0.5 mL•min^{-1} as detected by (**A**) electrical conductivity (EC) and (**B**) ultraviolet absorbance at 254 nm. IC conditions: 3.6 mM sodium carbonate eluent; column temperature = 45 °C; injection loop volume = 1 mL.

Figure s1A shows the IC-EC chromatograph had no standalone peaks with a baseline ~500–1,000 µS•cm^{-1}, indicating incomplete anion separation throughout the 60-minute elution time. At 0.5 mL•min^{-1}, the peaks shifted to greater elution times by up to ~20 minutes, but the baseline

levels were similar, indicating separation was not improved at the lower flowrate. Figure s1B shows Cl-N-NO_2^- eluted ~52–58 minutes at 0.7 mL•min^{-1} and 75–81 minutes at 0.5 mL•min^{-1}. While separation from the prior eluting peak was ~5 minutes at 0.5 mL•min^{-1} compared to ~2 minutes at 0.7 mL•min^{-1}, Figure s1A indicates the conductivity prior to Cl-N-NO_2^- elution was ~1,000 µS•cm^{-1} regardless of the flowrate. As such, an eluent flowrate of 0.7 mL•min^{-1} was selected for isolation to favor chromatographic throughput while manipulating other IC method conditions to improve separation. Figure s2 shows the impact of Na_2CO_3 eluent strength on Cl-N-NO_2^- separation at the recommended eluent strength of 3.6 mM compared to 1.8 and 7.2 mM.

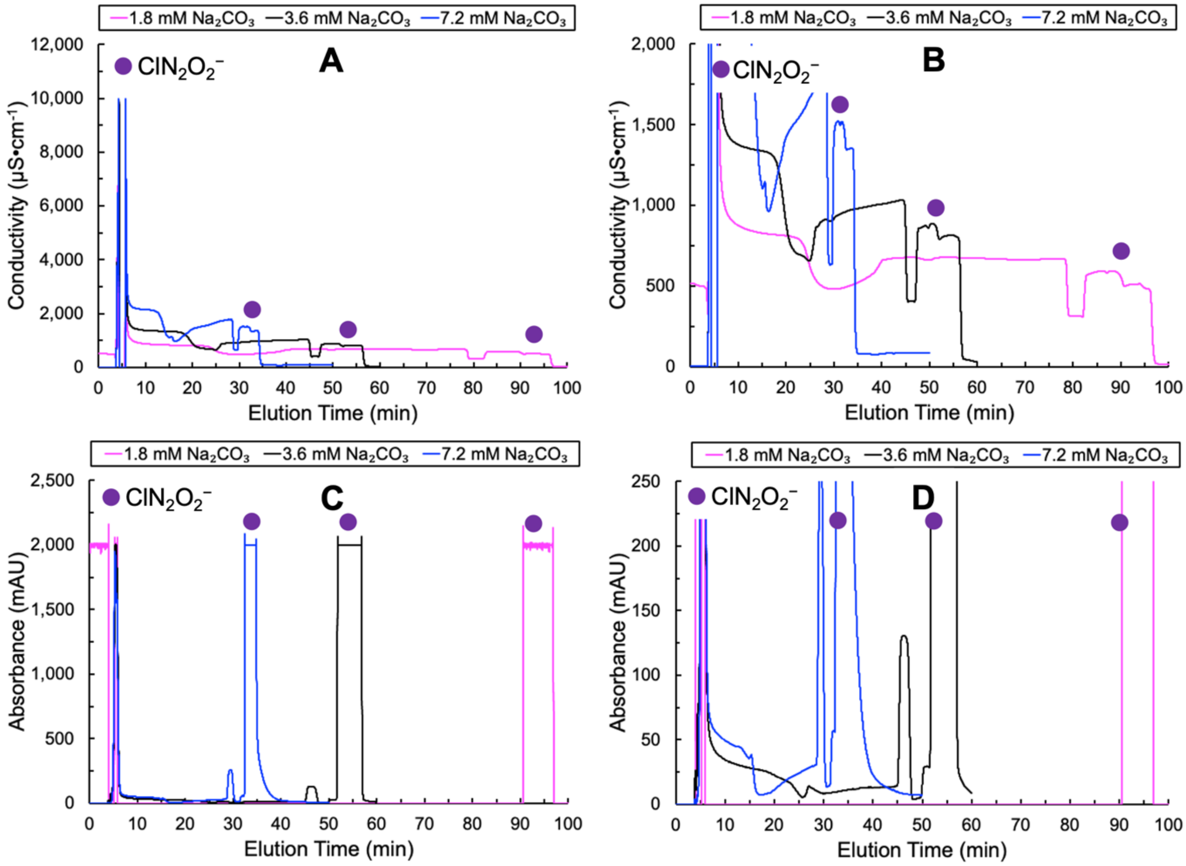

Figure s2. Ion chromatography (IC) method development for Cl-N-NO_2^- isolation from common anions showing the impact of Na_2CO_3 eluent strength on Cl-N-NO_2^- elution ~32–39 minutes at 7.2 mM, ~50–60 minutes at 3.6 mM, and ~90–96 minutes at 1.8 mM as detected by (**A**) electrical conductivity (EC), (**B**) EC zoomed to highlight Cl-N-NO_2^- elution, (**C**) ultraviolet absorbance at 243 nm, and (**D**) ultraviolet absorbance at 243 nm zoomed to highlight Cl-N-NO_2^- elution. IC conditions: eluent flowrate = 0.7 mL•min^{-1}; column temperature = 45 °C; injection loop volume = 1 mL.

Comparison of Figures s2A and s2C indicates the IC-EC baseline was ~500–1,000 µS•cm^{-1} prior to Cl-N-NO_2^- elution, indicating incomplete separation. The Cl-N-NO_2^- elution time increased with decreasing eluent strength from ~35 minutes with 7.2 mM Na_2CO_3 to ~95 minutes with 1.8 mM Na_2CO_3. However, as the IC-EC baseline did not approach zero prior to Cl-N-NO_2^- elution, the recommended 3.6 mM Na_2CO_3 eluent was used for isolation. Figure s3 shows the impact of injection loop size on Cl-N-NO_2^- separation.

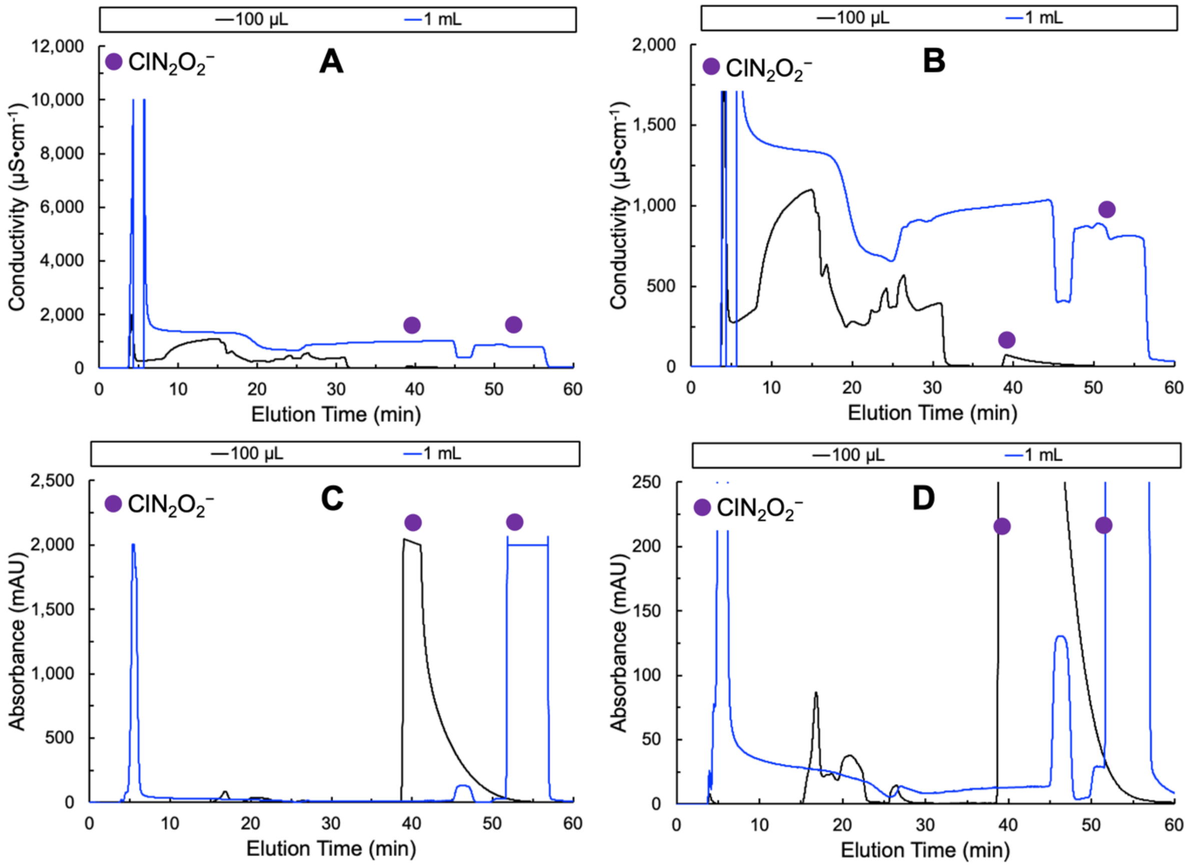

Figure s3. Ion chromatography (IC) method development for Cl-N-NO_2^- isolation from common anions showing the impact of IC injection loop size on Cl-N-NO_2^- elution ~38–50 minutes with the 100 µL loop and ~50–60 minutes with the 1 mL loop as detected by (**A**) electrical conductivity (EC), (**B**) EC zoomed to highlight Cl-N-NO_2^- elution, (**C**) ultraviolet absorbance at 243 nm (UV_{243}), and (**D**) UV_{243} zoomed to highlight Cl-N-NO_2^- elution. IC conditions: eluent flowrate = 0.7 mL•min^{-1}; eluent 3.6 mM Na_2CO_3; column temperature = 45 °C.

For the 1 mL loop, the IC-EC baseline varied between ~500–1,000 µS•cm^{-1} (Figure s3A) prior to Cl-N-NO_2^- elution at ~52–57 minutes (Figure s3C), indicating incomplete anion separation. In contrast, with the 100 µL injection loop, the baseline was near zero at ~32–38 minutes (Figure s3B) prior to Cl-N-NO_2^- elution at ~38–50 minutes (Figure s3D). Therefore, the 100 µL loop was selected for Cl-N-NO_2^- separation from the mixtures containing great concentrations of common anions.

The UV detector wavelength was set to 243 nm to match the Cl-N-NO_2^- peak molar absorptivity,[1] and a 2 nm bandwidth was used throughout. The mixtures formulated using the

NH_2Cl–NO_2^- reaction[1] had a Cl-N-NO_2^- UV_{243} signal that reached the detector maximum of 2,000 mAU within 0.5 minutes of the peak front and remained at 2,000 mAU for 4–8 minutes prior to gradually decreasing over the subsequent 10 to 15 minutes (Figure s4).

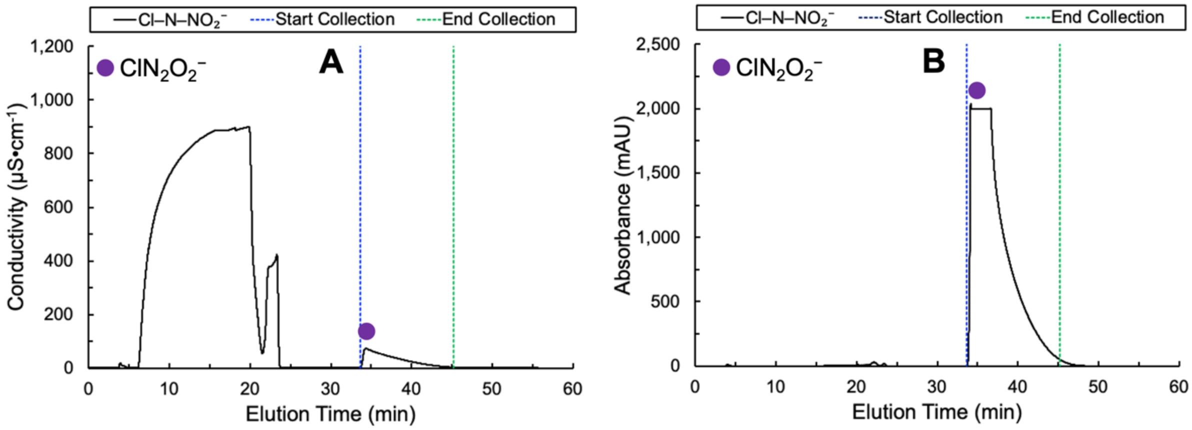

Figure s4. Ion chromatography (IC) method development for Cl-N-NO_2^- isolation from common anions showing Cl-N-NO_2^- elution ~34–45 minutes and the collection window as detected by (**A**) electrical conductivity (EC) and (**B**) ultraviolet absorbance at 243 nm (UV_{243}). The IC effluent was collected starting at the dotted blue line at ~34 minutes and ended at the dotted green line at ~ 45 minutes after the UV_{243} signal dropped below 50 mAU. IC conditions: eluent flowrate = 0.7 mL•min^{-1}; eluent 3.6 mM Na_2CO_3; column temperature = 45 °C; injection loop volume = 100 µL.

The Cl-N-NO_2^- reference material was collected starting at the peak front at ~34 minutes (i.e., vertical blue line in Figure s4A), continuing through the peak apex, and was ended after the IC-UV_{243} signal dropped below 50 mAU at ~45 minutes (i.e., vertical green line in Figure s4B). The IC isolation method conditions are summarized in Table s1.

Table s1. Metrohm 850 Professional Ion chromatography electrical conductivity (IC-EC) and ultraviolet absorbance at 243 nm (IC-UV_{243}) methods for chloronitramide anion (Cl-N-NO_2^-) isolation from mixtures formulated using the NH_2Cl–NO_2^- reaction detailed in prior work[1] and Cl-N-NO_2^- quantitation in this study.

Parameter	Units	Isolation	Quantitation
Sample Loop Size	–	100 µL	5 mL
Sample Volume	mL	10	10
Column Type	NA	Metrosep A Supp 7–250/4.0[a]	Metrosep A Supp 7–250/4.0[b]
Temperature	°C	50	45
Run Time	min	60	35
Eluent Flowrate	mL•min^{-1}	0.7	0.7
Eluent Type	NA	Na_2CO_3	Na_2CO_3
Eluent Strength	mM	3.6	14.4
Eluent Conductivity	µS•cm^{-1}	875	2,900
UV Detector Bandwidth	nm	2	2

[a] 3-year-old column with >10,000 injections
[b] new column purchased for this study

S1.1.3 Quantitation Method. The method development for Cl-N-NO_2^- quantitation was based on the IC isolation parameters with the standards prepared in 1 mM borate buffer at pH 9. A 5 mL injection loop, the largest recommended by the manufacturer, was used to achieve the lowest possible method detection limit. The column temperature was set at the recommended 45 °C to maximize the column lifetime. The eluent flowrate was kept at 0.7 mL•min^{-1}, same as the isolation method, but the eluent strength was increased to 14 mM to decrease the Cl-N-NO_2^- elution time and increase sampler throughput without sacrificing separation. A UV wavelength of 243 nm was selected for quantitation to match the Cl-N-NO_2^- molar absorptivity maxima[1] and the bandwidth was set at 2 nm, the default setting for the detector. The Cl-N-NO_2^- IC-EC and IC−UV_{243} peaks were defined by their start, apex, and endpoint. The area under the curve and peak height were used for quantitation. For IC-EC, the baselines around Cl-N-NO_2^- elution were flat; as such, the peak start and end were taken to be 0.5 minutes prior to the peak start and following the peak end. For IC-UV_{243}, the presence of baseline noise necessitated the peak start

and end to be taken as the start of the peak front and endpoint of the signal, without the 0.5 minutes on either side. Table s1 summarizes the conditions of the Cl-N-NO_2^- IC method.

S1.2 Chloronitramide Anion Standard Curve Preparation

Cl-N-NO_2^- reference materials were prepared in amber glass bottles and standardized using a Shimadzu UV-2450 UV-Vis spectrophotometer. IC-isolates were generated as detailed in Section 2.2 and the Supporting Information S1.1.2 and diluted up to 10X in 1 mM borate buffer at pH 9 such that their absorbance at 243 nm (A_{243}) was 0.2–0.8 with a 1 cm quartz cuvette. The reference material concentration was calculated using its previously determined molar absorptivity,[1] $\varepsilon_{243} = 5{,}310\ M^{-1}{\bullet}cm^{-1}$, and the associated dilution factor. A stock solution was formulated at nominal concentration of 2,000 $\mu g{\bullet}L^{-1}$ (Stock A) and the actual concentration determined by measuring the A_{243} with a 1 cm quartz cuvette. The Stock A concentration was remeasured daily prior to formulating Cl-N-NO_2^- standard curves. IC standards were made using volumetric dilutions of Stock A at concentrations of 500, 400, 300, 250, 200, 150, 125, 100, 75, 50, 25, 15, 10, and 5 $\mu g{\bullet}L^{-1}$. Standards were run by IC and the peak area and peak height for conductivity and UV_{243} were linearly regressed to determine a slope and intercept. A standard curve was run with each batch of samples, every 1–2 days depending on the number of samples in each batch.

S1.3 Chloronitramide Anion MDL, LOD, and LOQ Determinations

The IC-EC and IC-UV_{243} MDLs were determined following the EPA procedure[2] using a minimum of seven blanks consisting of lab-grade water amended with 1 mM borate buffer to determine the blank MDL (MDL_b) and a minimum of seven samples of lab-grade water amended with 1 mM borate buffer and 25 $\mu g{\bullet}L^{-1}$ Cl-N-NO_2^- to determine the sample MDL (MDL_s). A Cl-N-NO_2^- spike of 25 $\mu g{\bullet}L^{-1}$ was selected to produce a signal-to-noise ratio of 8–14, which was within the recommended range of 5–20. The resulting MDL for each detector was the greater of the MDL_b and MDL_s.[2] The MDL_b was calculated using Equation s1:

$$MDL_b = \bar{x}_b + t_{99,\,n-1} \cdot S_b \qquad \text{Equation s1}$$

In Equation s1, $\bar{x}_b$ is the mean area under the curve of the seven blanks, S_b is the corresponding standard deviation, and $t_{99,\,n-1} = 2.998$, is the t-value for a one-tailed 99th percentile t statistic with n−1 (i.e., 7) degrees of freedom. The MDL_s was calculated using Equation s2:

$$MDL_s = t_{99,\,n-1} \cdot S_s \qquad \text{Equation s2}$$

In Equation s2, $t_{99,\,n-1} = 2.998$ (i.e., same as in Equation s1) and S_S is the Cl-N-NO_2^- peak area standard deviation.

The LOD and LOQ were calculated using one method for peak area and another method for peak height. The same blanks and Cl-N-NO_2^- standard curves used in the MDL calculations were used for the LOD and LOQ calculations. For peak area, the LOD and LOQ were calculated using the blank determination method[3] and the LOD and LOQ were calculated[4] using Equations s3 and s4:

$$LOD = \bar{x}_b + 3 \cdot S_b \qquad \text{Equation s3}$$

$$LOQ = \bar{x}_b + 10 \cdot S_b \qquad \text{Equation s4}$$

In Equations s3 and s4, $\bar{x}_b$ is the mean of the blank response and S_b is the corresponding standard deviation.

For peak height, the LOD and LOQ were calculated using the signal-to-noise ratio (S/N), which is commonly used with signals that contain baseline noise.[3] The S/N was calculated using Equation s5:

$$S/N = \frac{2H}{h} \qquad \text{Equation s5}$$

In Equation s5, H is the height of the peak measured from the maximum of the peak from the extrapolated baseline and h is the peak-to-peak background noise observed over a distance equal to 20-times the width at a half-height of the peak centered on the analyte elution time. LOD and

LOQ are then calculated using a S/N of 3:1 and 10:1, respectively, and solving for H which is then converted to a concentration using a standard curve.

S1.4 Water Sample Collection for Matrix Testing

Mini coolers with sample bottles were shipped to eleven residential homes across the U.S. for tap water collection. Prior to sample collection, cold water faucets were flushed at full flow for 5 minutes to mitigate stagnation effects and capture water from the distribution system main. Tap water samples were collected in two 250 mL HDPE bottles, filled headspace-free with cold water, capped firmly, and covered in bubble wrap for return shipping. The water samples were shipped overnight to the University of Arkansas and refrigerated at 4 °C. Samples were tested for pH, total chlorine, monochloramine, and $Cl\text{-}N\text{-}NO_2^-$. All samples were tested within 10 days of one another and sample collection and thus the resultant $Cl\text{-}N\text{-}NO_2^-$ concentrations may be greater than the actual concentrations due to NH_2Cl decomposition during storage.

S1.5 Chloronitramide Anion Reproducibility and Matrix Testing

ASTM D8272[5] was followed to assess the reproducibility and repeatability (i.e., matrix testing) of $Cl\text{-}N\text{-}NO_2^-$ quantitation by IC-EC and IC-UV_{243}. The reproducibility testing used 1 mM borate buffer at pH 9 spiked with four $Cl\text{-}N\text{-}NO_2^-$ standards (15, 25, 50, and 100 $\mu g \bullet L^{-1}$) and run in triplicate on seven different days. The IC system was power cycled daily prior to running the reproducibility samples.

The repeatability testing consisted of triplicate measurements of four $Cl\text{-}N\text{-}NO_2^-$ standards (15, 25, 50, and 100 $\mu g \bullet L^{-1}$) spiked into twelve water matrices which included (i) synthetic groundwater and (ii) eleven tap water samples collected from eight chloramine utilities, including one Pennsylvania (PA), three in Texas (TX-1, TX-2, and TX-3), one in South Carolina (SC),

one in California (CA), one in Oklahoma (OK), and one in Minnesota (MN); and three free chlorine utilities including one in Arkansas (AR) and two in Georgia (GA-1 and GA-2). Standard curves were run with each water type to quantify $Cl\text{-}N\text{-}NO_2^-$ by IC-EC and IC-UV_{243}.

S1.6 Chloronitramide Anion Quantitation by HILIC–UHRMS

The HILIC–UHRMS $Cl\text{-}N\text{-}NO_2^-$ method used a Vanquish liquid chromatograph (Thermo) coupled to a QExactive HF orbitrap mass spectrometer (Thermo). A SeQuant ZIC-pHILIC column (5 µm, 150 × 2.1 mm) with guard column (20 × 2.1 mm) was installed and operated at a flow rate of 0.15 $mL \cdot min^{-1}$. A gradient was run using two solvents: (A) 7.5:92.5 % water:acetonitrile with 10 mM ammonium acetate and (B) 85:15 % water:acetonitrile with 10 mM ammonium acetate. The gradient consisted of a 5-minute hold at 100 % A; A was decreased over 4 minutes to 90 % then over 6 minutes to 0 % and held at 0 % for 5 minutes before returning to the starting conditions, which were held for 10 minutes before the next injection. Selected ion monitoring was performed using a window of m/z = 94–98, an injection time of 10 ms, a resolution of 240k, and an AGC target of 1×10^5 ions. Calibration standards were prepared and analyzed at the beginning of each run and had 50 $\mu g \cdot L^{-1}$ of a ^{15}N labelled $Cl\text{-}N\text{-}NO_2^-$ internal standard added, prepared as described previously.[1] The internal standard addition allowed for correction of matrix effects. Lastly, the ^{35}Cl:^{37}Cl ion ratio was used for quality control to confirm the presence of $Cl\text{-}N\text{-}NO_2^-$ and absence of any co-eluting interferents.

S2. Results and Discussion

S2.1 MDL, LOD, and LOQ

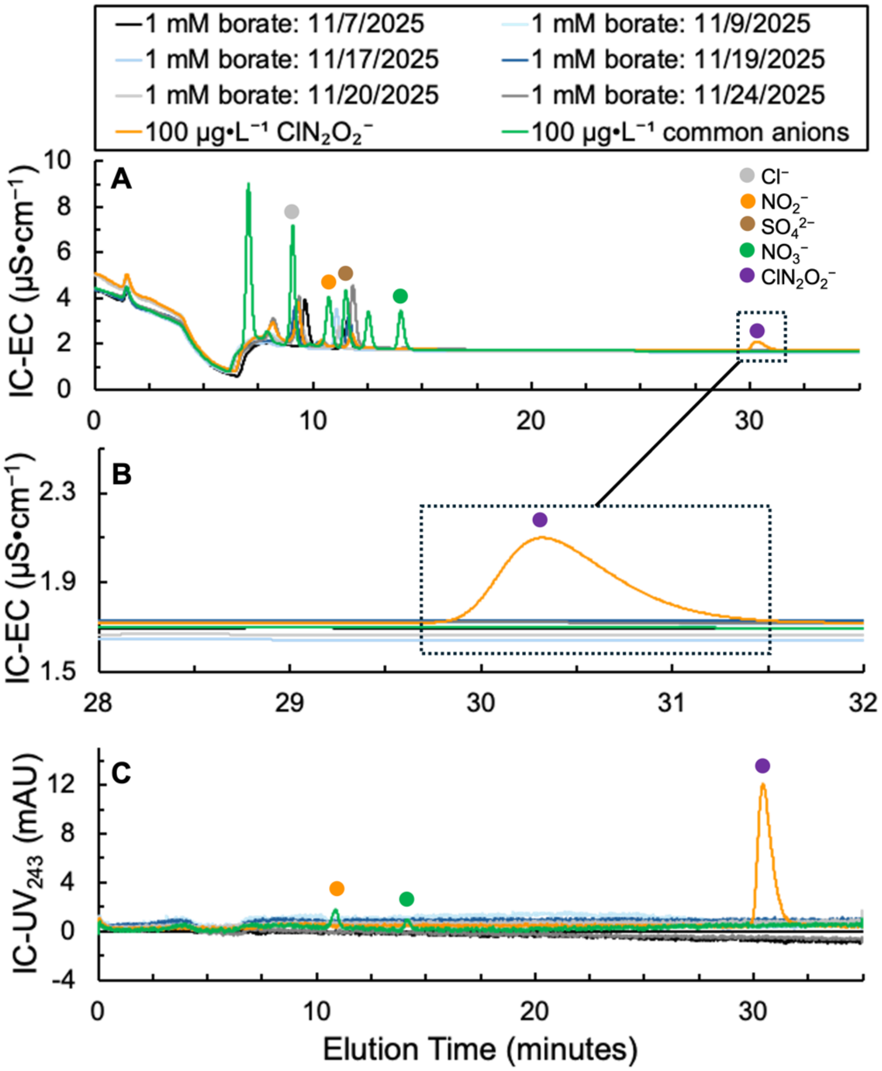

Figure s5. Ion chromatographs for six 1 mM borate buffer samples at pH 9, a 100 µg•L^{-1} common anion standard, and a 100 µg•L^{-1} Cl-N-NO_2^- standard prepared in 1 mM borate buffer at pH 9. Cl-N-NO_2^- elution occurs ~30 minutes as detected by (**A**) electrical conductivity (EC) showing the entire elution period (0–35 minutes), (**B**) EC zoomed to highlight the baseline signals around Cl-N-NO_2^- elution, and (**C**) ultraviolet absorbance at 243 nm (UV_{243}). Six 1 mM borate buffer samples show peaks detected by IC-EC between ~7–15 minutes corresponding to the approximate elution times of common anions; a 100 µg•L^{-1} common anion standard is shown for comparison. IC method conditions: eluent flowrate = 0.7 mL•min^{-1}; eluent strength and type = 3.6 mM Na_2CO_3; column temperature = 45 °C; injection loop volume = 5 mL.

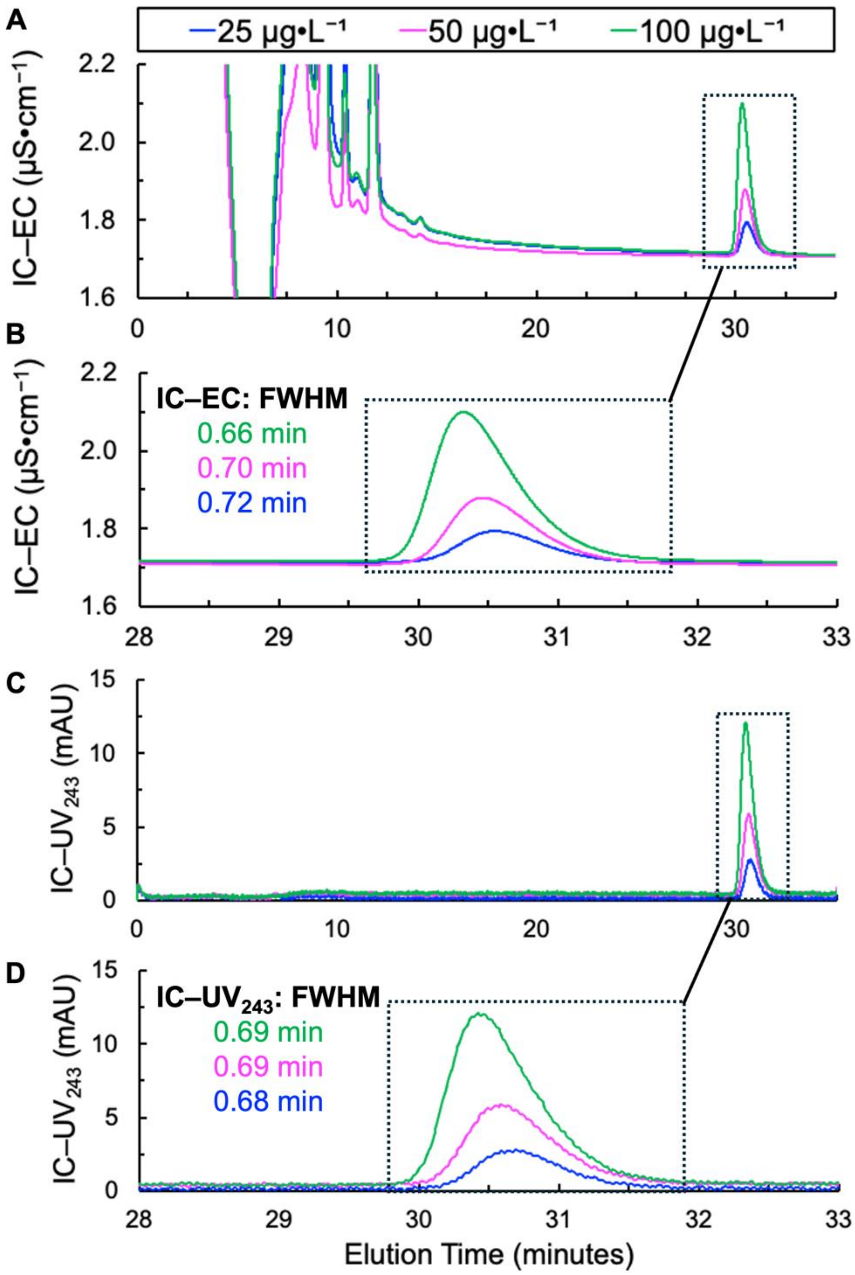

Figure s6. Ion chromatographs of 25, 50, and 100 μg•L^{-1} Cl-N-NO_2^- standard prepared in 1 mM borate buffer at pH 9 as detected by (**A**) electrical conductivity (EC) showing the entire elution period (0–35 minutes), (**B**) EC zoomed to highlight the standard signals around Cl-N-NO_2^- elution, and (**C**) ultraviolet absorbance at 243 nm (UV_{243}) showing the entire elution period (0–35 minutes), and (**D**) UV_{243} zoomed to highlight the standard signals around Cl-N-NO_2^- elution. The Cl-N-NO_2^- full-width at half maximum (FWHM) values are listed in panels (B) and (D), color-coded by their concentration.

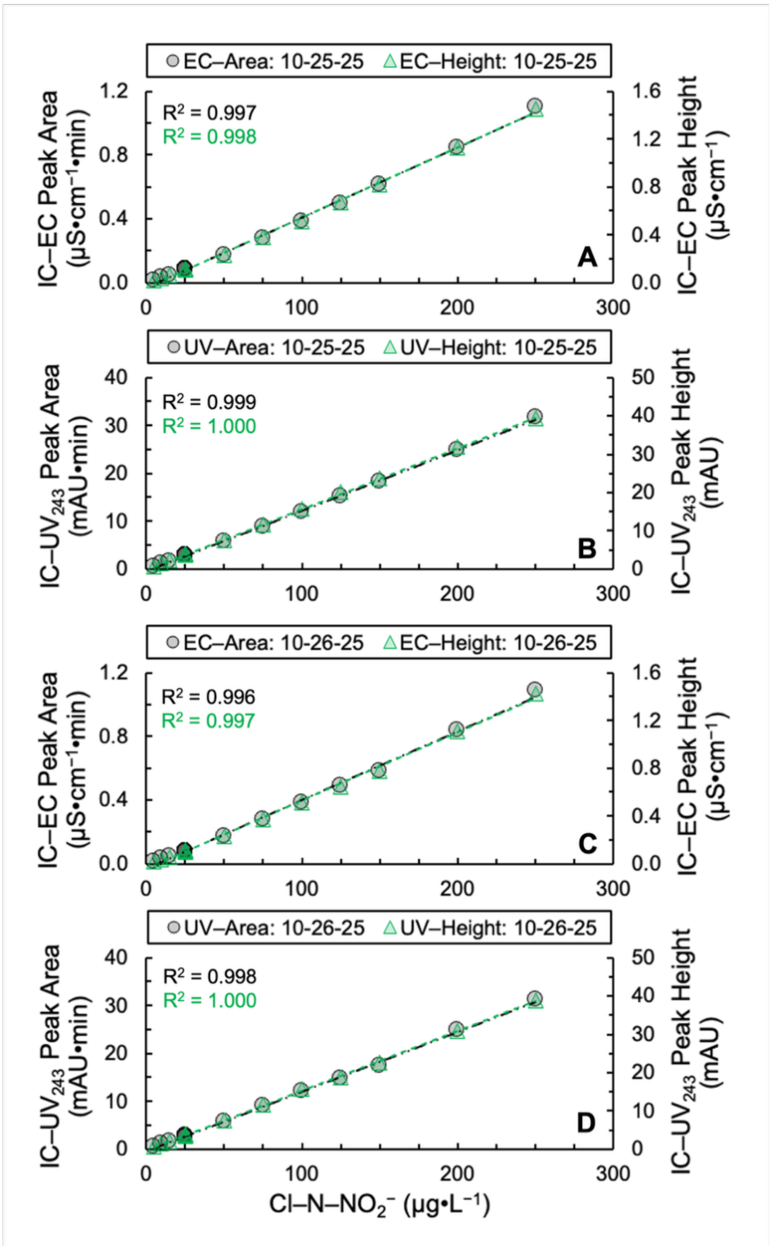

Figure s7. Ion chromatography (IC) Cl-N-NO_2^- standard curves with correlation coefficients (R^2) quantified by (**A**) electrical conductivity (EC) by Peak Area (black points and line) and Peak Height (green points and line) for 10-25-25, (**B**) ultraviolet absorbance at 243 nm (UV_{243}) by Peak Area (black points and line) and Peak Height (green points and line) for 10-25-25, (**C**) EC by Peak Area (black points and line) and Peak Height (green points and line) for 10-26-25, and (**D**) UV_{243} by Peak Area (black points and line) and Peak Height (green points and line) for 10-26-25. Lines are the best-fit linear regression of the standard curves.

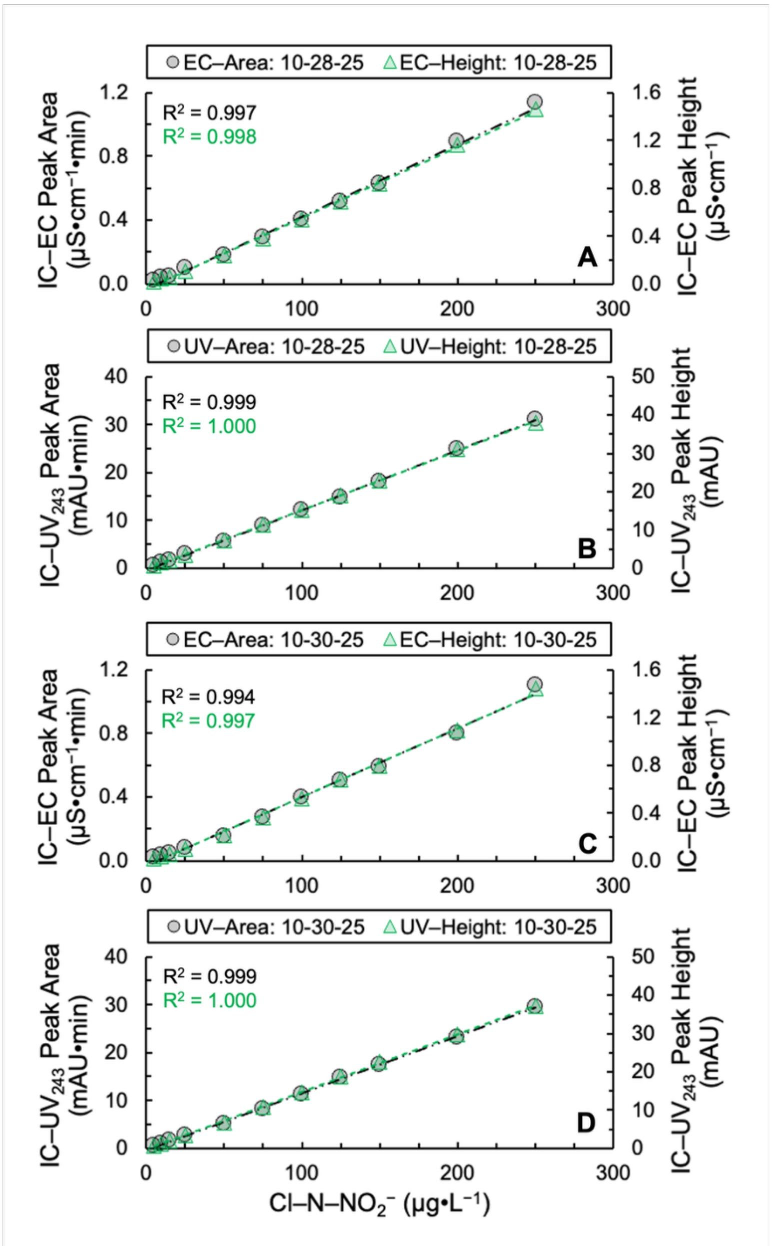

Figure s8. Ion chromatography (IC) Cl-N-NO_2^- standard curves with correlation coefficients (R^2) quantified by (**A**) electrical conductivity (EC) by Peak Area (black points and line) and Peak Height (green points and line) for 10-28-25, (**B**) ultraviolet absorbance at 243 nm (UV_{243}) by Peak Area (black points and line) and Peak Height (green points and line) for 10-28-25, (**C**) EC by Peak Area (black points and line) and Peak Height (green points and line) for 10-30-25, and (**D**) UV_{243} by Peak Area (black points and line) and Peak Height (green points and line) for 10-30-25. Lines are the best-fit linear regression of the standard curves.

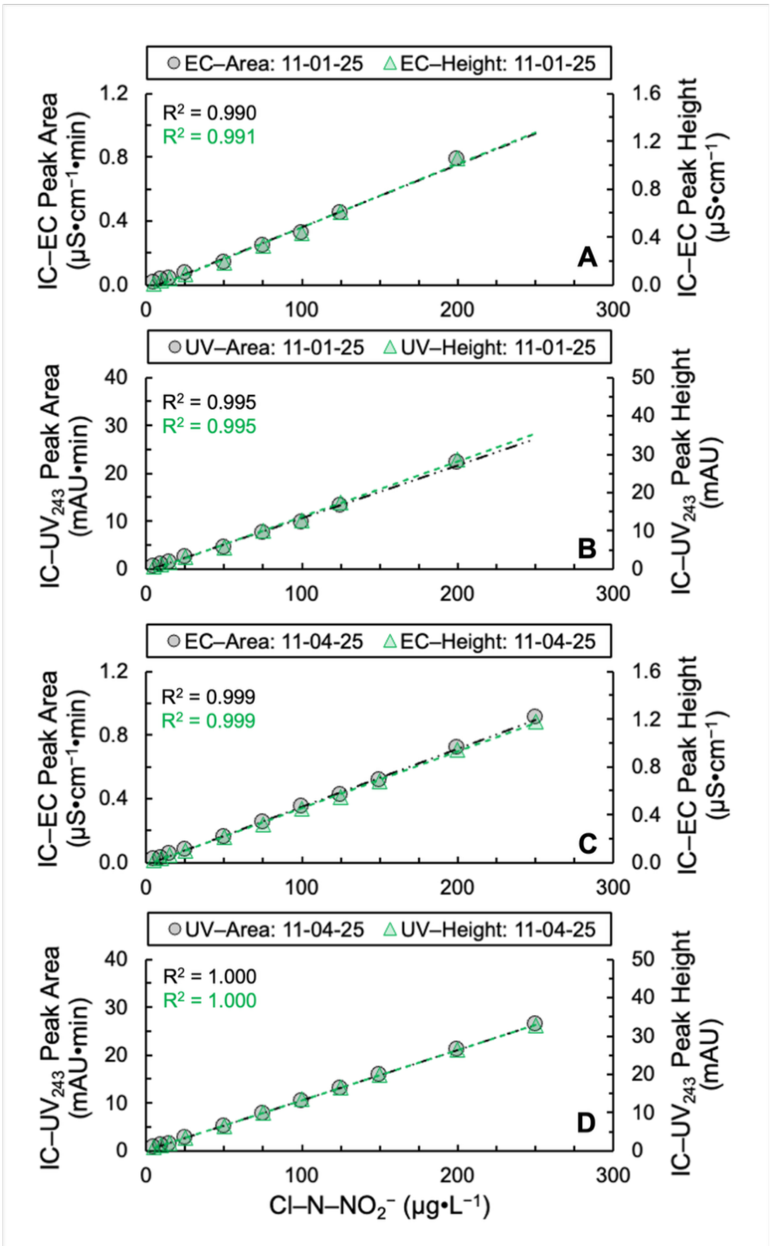

Figure s9. Ion chromatography (IC) Cl-N-NO_2^- standard curves with correlation coefficients (R^2) quantified by (**A**) electrical conductivity (EC) by Peak Area (black points and line) and Peak Height (green points and line) for 11-01-25, (**B**) ultraviolet absorbance at 243 nm (UV_{243}) by Peak Area (black points and line) and Peak Height (green points and line) for 11-01-25, (**C**) EC by Peak Area (black points and line) and Peak Height (green points and line) for 11-04-25, and (**D**) UV_{243} by Peak Area (black points and line) and Peak Height (green points and line) for 11-04-25. Lines are the best-fit linear regression of the standard curves.

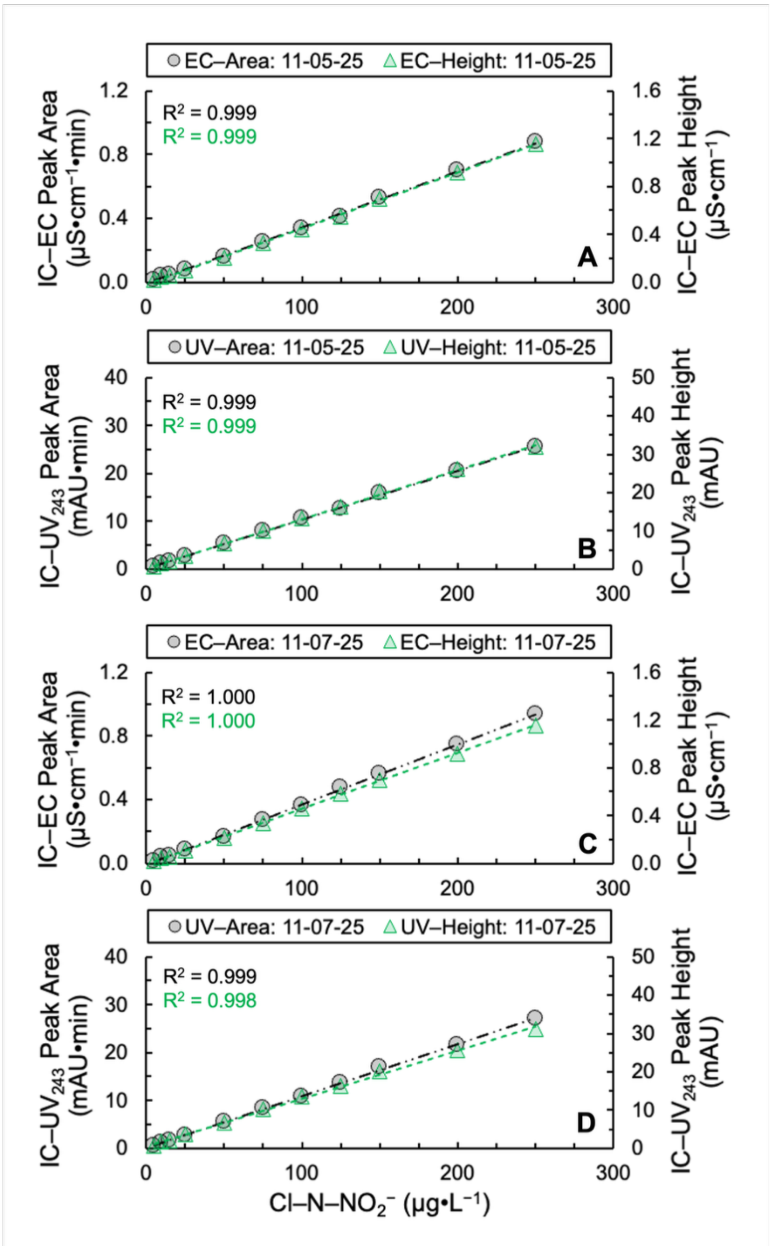

Figure s10. Ion chromatography (IC) Cl-N-NO_2^- standard curves with correlation coefficients (R^2) quantified by (**A**) electrical conductivity (EC) by Peak Area (black points and line) and Peak Height (green points and line) for 11-05-25, (**B**) ultraviolet absorbance at 243 nm (UV_{243}) by Peak Area (black points and line) and Peak Height (green points and line) for 11-05-25, (**C**) EC by Peak Area (black points and line) and Peak Height (green points and line) for 11-07-25, and (**D**) UV_{243} by Peak Area (black points and line) and Peak Height (green points and line) for 11-07-25. Lines are the best-fit linear regression of the standard curves.

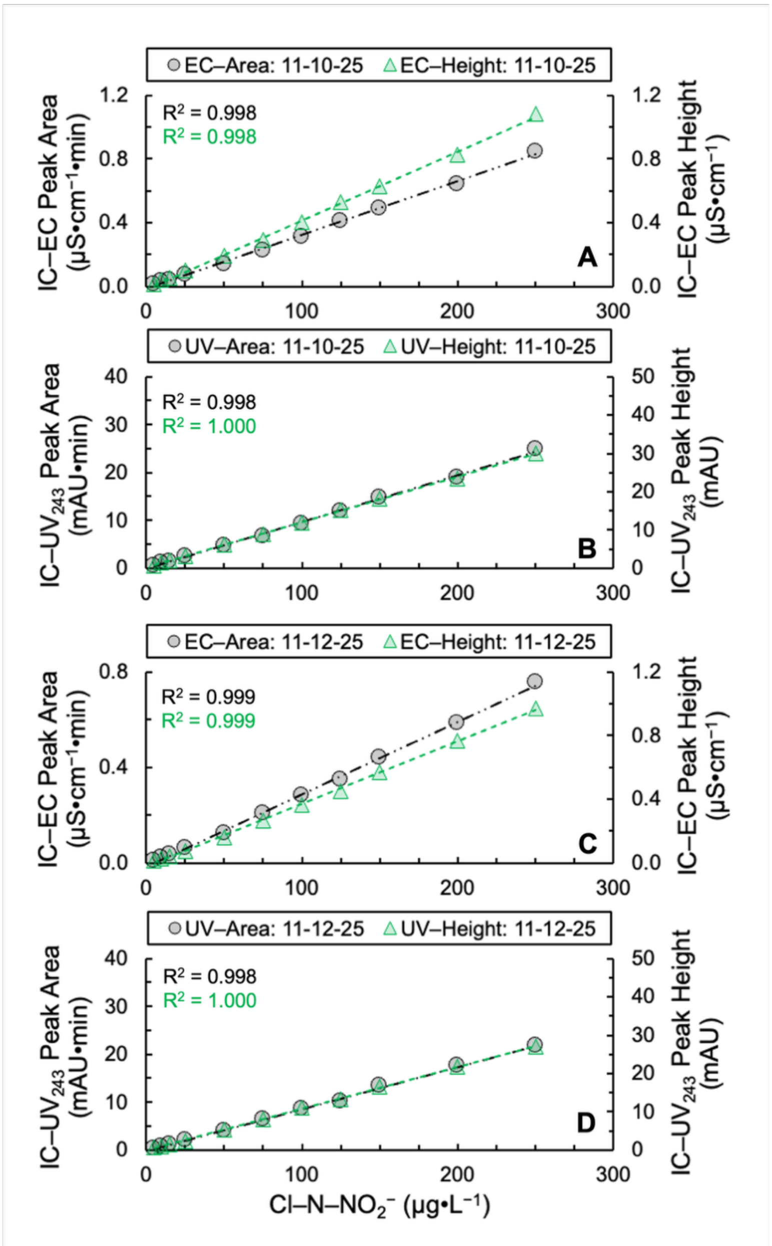

Figure s11. Ion chromatography (IC) Cl-N-NO_2^- standard curves with correlation coefficients (R^2) quantified by (**A**) electrical conductivity (EC) by Peak Area (black points and line) and Peak Height (green points and line) for 11-10-25, (**B**) ultraviolet absorbance at 243 nm (UV_{243}) by Peak Area (black points and line) and Peak Height (green points and line) for 11-10-25, (**C**) EC by Peak Area (black points and line) and Peak Height (green points and line) for 11-12-25, and (**D**) UV_{243} by Peak Area (black points and line) and Peak Height (green points and line) for 11-12-25. Lines are the best-fit linear regression of the standard curves.

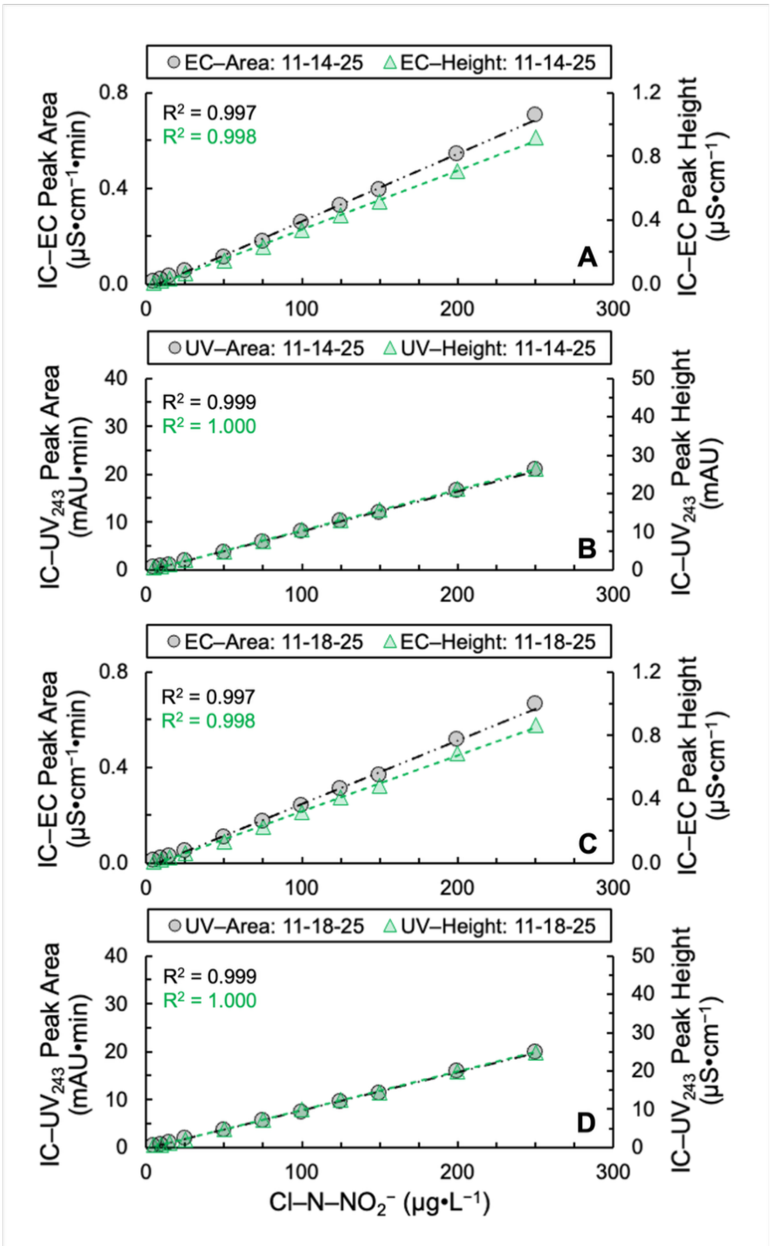

Figure s12. Ion chromatography (IC) Cl-N-NO_2^- standard curves with correlation coefficients (R^2) quantified by (**A**) electrical conductivity (EC) by Peak Area (black points and line) and Peak Height (green points and line) for 11-14-25, (**B**) ultraviolet absorbance at 243 nm (UV_{243}) by Peak Area (black points and line) and Peak Height (green points and line) for 11-14-25, (**C**) EC by Peak Area (black points and line) and Peak Height (green points and line) for 11-18-25, and (**D**) UV_{243} by Peak Area (black points and line) and Peak Height (green points and line) for 11-18-25. Lines are the best-fit linear regression of the standard curves.

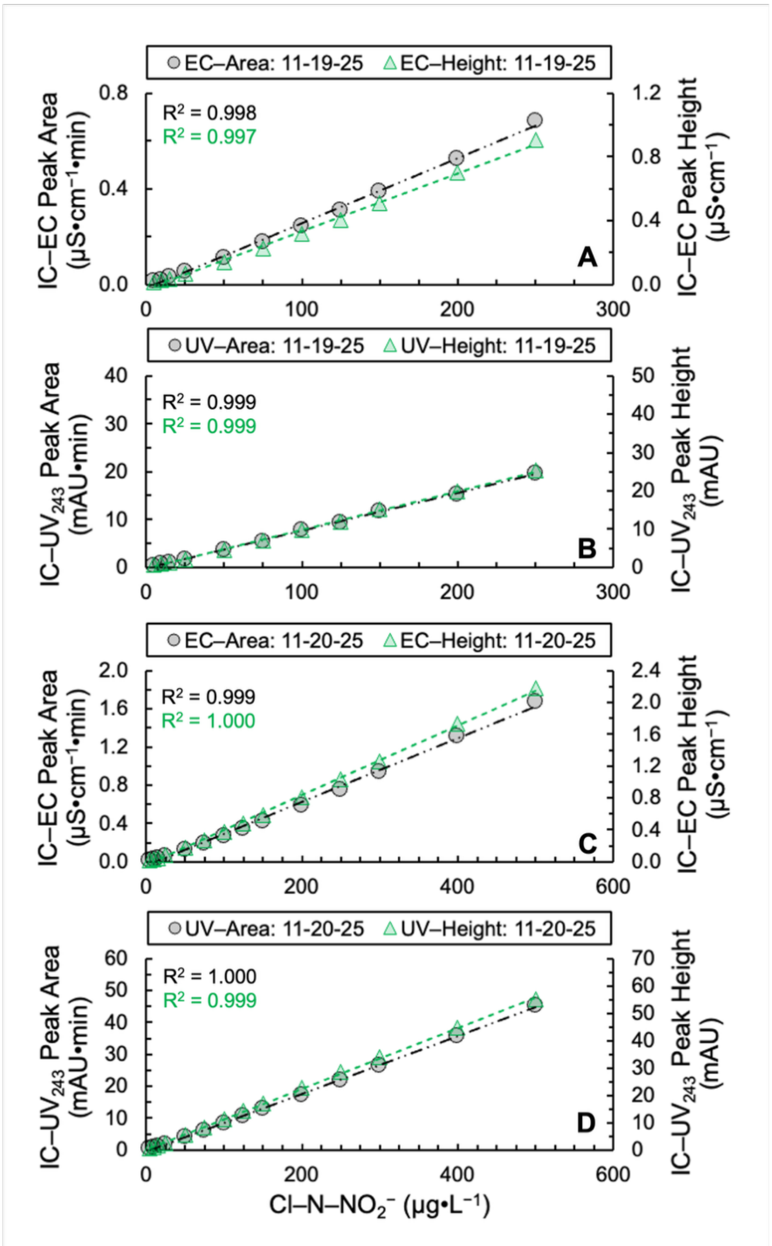

Figure s13. Ion chromatography (IC) Cl-N-NO_2^- standard curves with correlation coefficients (R^2) quantified by (**A**) electrical conductivity (EC) by Peak Area (black points and line) and Peak Height (green points and line) for 11-19-25, (**B**) ultraviolet absorbance at 243 nm (UV_{243}) by Peak Area (black points and line) and Peak Height (green points and line) for 11-19-25, (**C**) EC by Peak Area (black points and line) and Peak Height (green points and line) for 11-20-25, and (**D**) UV_{243} by Peak Area (black points and line) and Peak Height (green points and line) for 11-20-25. Lines are the best-fit linear regression of the standard curves.

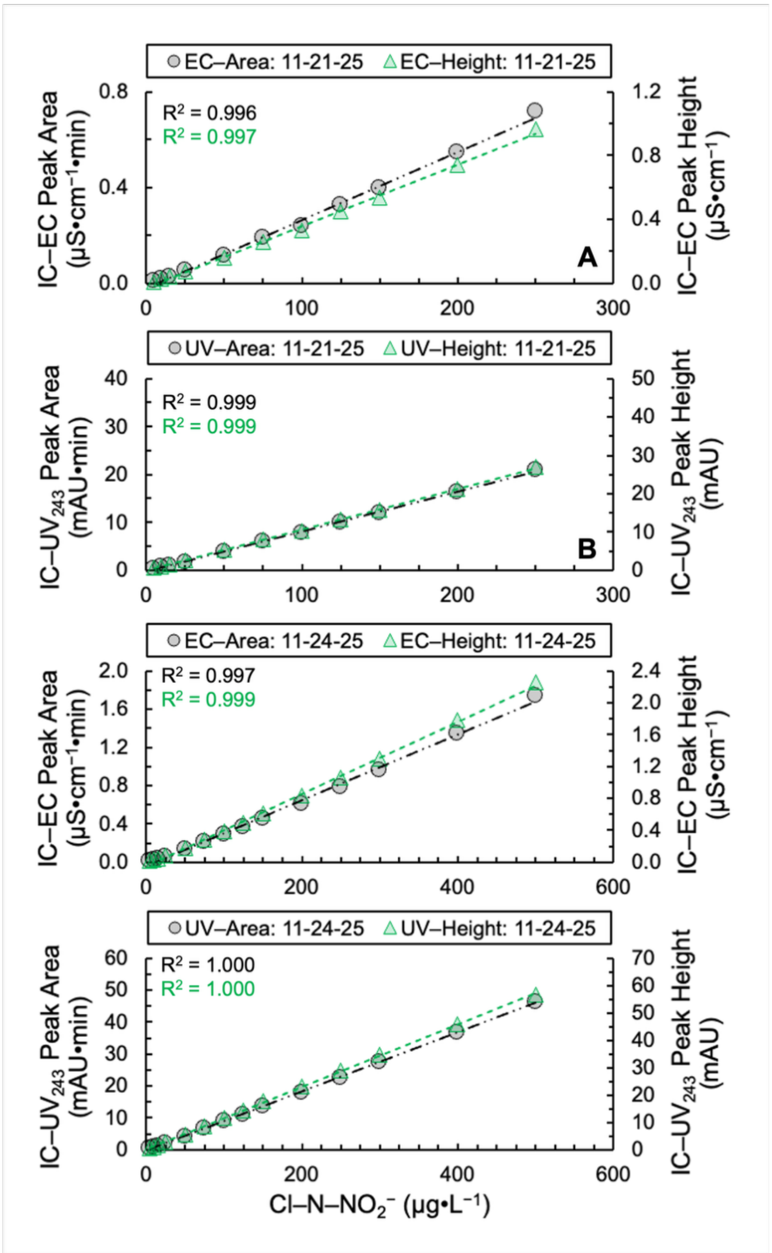

Figure s14. Ion chromatography (IC) Cl-N-NO_2^- standard curves with correlation coefficients (R^2) quantified by (**A**) electrical conductivity (EC) by Peak Area (black points and line) and Peak Height (green points and line) for 11-21-25, (**B**) ultraviolet absorbance at 243 nm (UV_{243}) by Peak Area (black points and line) and Peak Height (green points and line) for 11-21-25, (**C**) EC by Peak Area (black points and line) and Peak Height (green points and line) for 11-24-25, and (**D**) UV_{243} by Peak Area (black points and line) and Peak Height (green points and line) for 11-24-25. Lines are the best-fit linear regression of the standard curves.

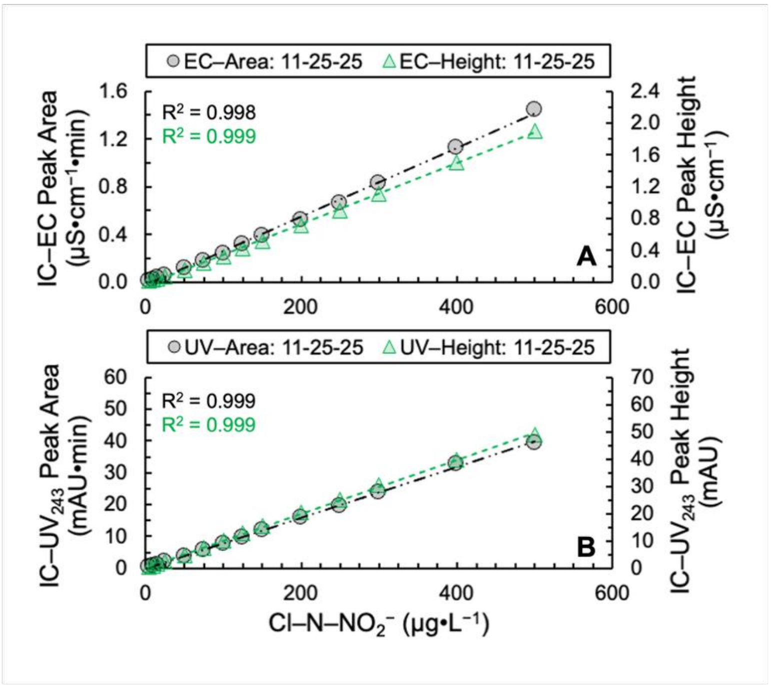

Figure s15. Ion chromatography (IC) Cl-N-NO_2^- standard curves with correlation coefficients (R^2) quantified by (**A**) electrical conductivity (EC) by Peak Area (black points and line) and Peak Height (green points and line) for 11-25-25 and (**B**) ultraviolet absorbance at 243 nm (UV_{243}) by Peak Area (black points and line) and Peak Height (green points and line) for 11-25-25. Lines are the best-fit linear regression of the standard curves.

Table s2. Ion chromatography–electrical conductivity (IC-EC) and ion chromatography–ultraviolet absorbance at 243 nm ($IC\text{-}UV_{243}$) chloronitramide anion ($Cl\text{-}N\text{-}NO_2^-$) peak area method detection level by blank (Peak Area MDL_b, lab-grade water) and sample (Peak Area MDL_s, lab-grade water spiked with $Cl\text{-}N\text{-}NO_2^-$ at 25 µg•L^{-1}) used to determine the $Cl\text{-}N\text{-}NO_2^-$ MDL for IC-EC and $IC\text{-}UV_{243}$ and the limit of detection (LOD) and limit of quantitation (LOQ) for $IC\text{-}UV_{243}$ using the Slope and Y-Intercept from the standard curves from 10-25-25 shown in Figure s7.

		Peak Area		Peak Height	
Sample	$Cl\text{-}N\text{-}NO_2^-$ (µg•L^{-1})	IC-EC (µS•cm^{-1}•min)	$IC\text{-}UV_{243}$ (mAU•min)	IC-EC (µS•cm^{-1})	$IC\text{-}UV_{243}$ (mAU)
Blank 1	0	0.002	0.075	0.003	0.181
Blank 2	0	0.002	0.098	0.003	0.181
Blank 3	0	0.002	0.011	0.003	0.276
Blank 4	0	0.002	0.013	0.003	0.174
Blank 5	0	0.002	0.046	0.003	0.168
Blank 6	0	0.003	0.099	0.004	0.193
Blank 7	0	0.003	0.056	0.003	0.182
Blank 8	0	0.002	0.023	0.003	0.278
S_b	–	0.000	0.036	0.000	0.046
$\bar{x}_b$	–	0.002	0.053	0.003	0.204
h	–			0.003	0.204
Sample 1	25	0.082	2.664	0.108	3.628
Sample 2	25	0.088	2.802	0.115	3.837
Sample 3	25	0.088	2.804	0.116	3.795
Sample 4	25	0.088	2.919	0.114	3.880
Sample 5	25	0.077	2.529	0.103	3.463
Sample 6	25	0.084	2.745	0.111	3.745
Sample 7	25	0.082	2.944	0.111	3.945
Sample 8	25	0.086	2.756	0.113	3.749
S_s	–	0.004	0.134	0.004	0.152
H	–	–	–	0.111	3.755
$t_{99, n-1}$	–	2.998	2.998	2.998	2.998
Peak Area MDL_b	–	0.004	0.160	0.004	0.341
Peak Area MDL_s	–	0.012	0.400	0.013	0.455
Controlling Factor	–	MDL_s	MDL_s	MDL_s	MDL_s
MDL (µg•L^{-1})	–	9.3	6.2	8.7	4.1
LOD (µg•L^{-1})	–	7.4	5.8	3.1	5.5
LOQ (µg•L^{-1})	–	8.2	8.3	3.2	11.2

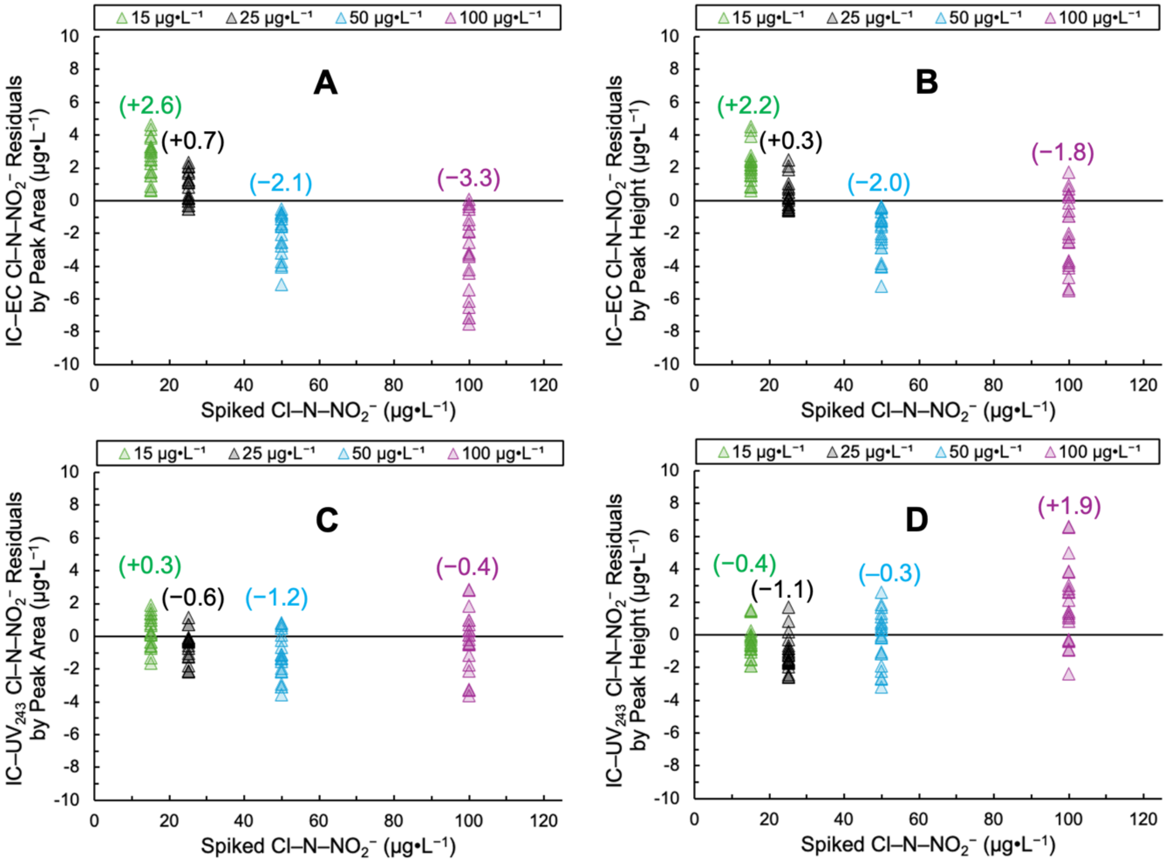

Figure s16. Ion chromatography (IC) electrical conductivity (EC) and IC–ultraviolet absorbance at 243 nm (UV_{243}) reproducibility testing with $Cl\text{-}N\text{-}NO_2^-$ spiked at 15, 25, 50, and 100 μg•L^{-1} in 1 mM borate buffer at pH 9. (**A**) IC-EC measured by peak area, (**B**) IC-EC measured by peak height, (**C**) IC-UV_{243} measured by peak area, (**D**) IC-UV_{243} measured by peak height. The average residual, $\overline{r_i}$, calculated as the total sum of residuals divided by number of observations (n = 21) in μg•L^{-1} is shown in parentheses, color-coded based on the $Cl\text{-}N\text{-}NO_2^-$ concentration.

Table s3. Water characterization for the tap waters used in the matrix testing.

Water	pH	Total Chlorine [d]	Monochloramine [e]	Free Chlorine [f]
AR [a]	7.4	0.4	--	0.4
PA [a]	7.7	1.4	1.2	--
TX-1 [a]	7.4	0.2	0.1	--
TX-2 [a]	7.7	2.2	1.9	--
GA-1 [a]	8.3	0.3	--	0.3
GA-2 [a]	8.0	2.4	--	2.4
SC [a]	7.3	1.4	1.2	--
CA [a]	7.7	--	--	--
GW [b]	8.1	--	--	--
TX-3 [a]	7.7	3.0	2.7	--
OK [a]	8.8	1.6	1.4	--
MN [a]	7.9	2.5	2.3	--

[a] state abbreviation: AR – Arkansas; PA – Pennsylvania; TX – Texas; GA – Georgia; SC – South Carolina; CA – California; OK – Oklahoma; MN - Minnesota

[b] Synthetic groundwater

[d] Total chlorine by DPD[6] in mg•L^{-1} as Cl_2

[e] Monochloramine by Indophenol[7] in mg•L^{-1} as Cl_2

[f] Free chlorine by DPD[6] in mg•L^{-1} as Cl_2

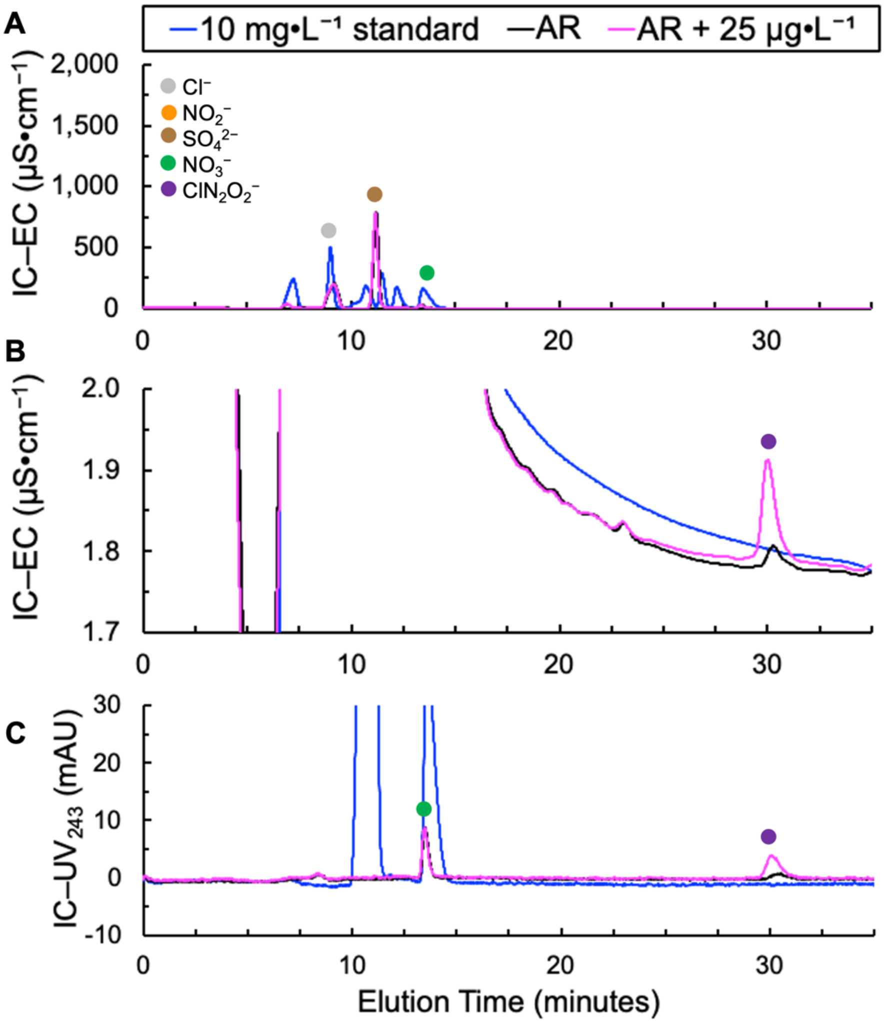

Figure s17. Ion chromatography (IC) chromatograms of a 10 mg•L^{-1} common anion standard, the AR tap water sample, and the AR tap water sample spiked with 25 µg•L^{-1} Cl-N-NO_2^-. (**A**) electrical conductivity (IC-EC), (**B**) IC-EC zoomed in to show the Cl-N-NO_2^- peaks, and (**C**) ultraviolet absorbance at 243 nm (IC-UV_{243}). The colored dots above the peaks indicate the anions detected in the tap water samples.

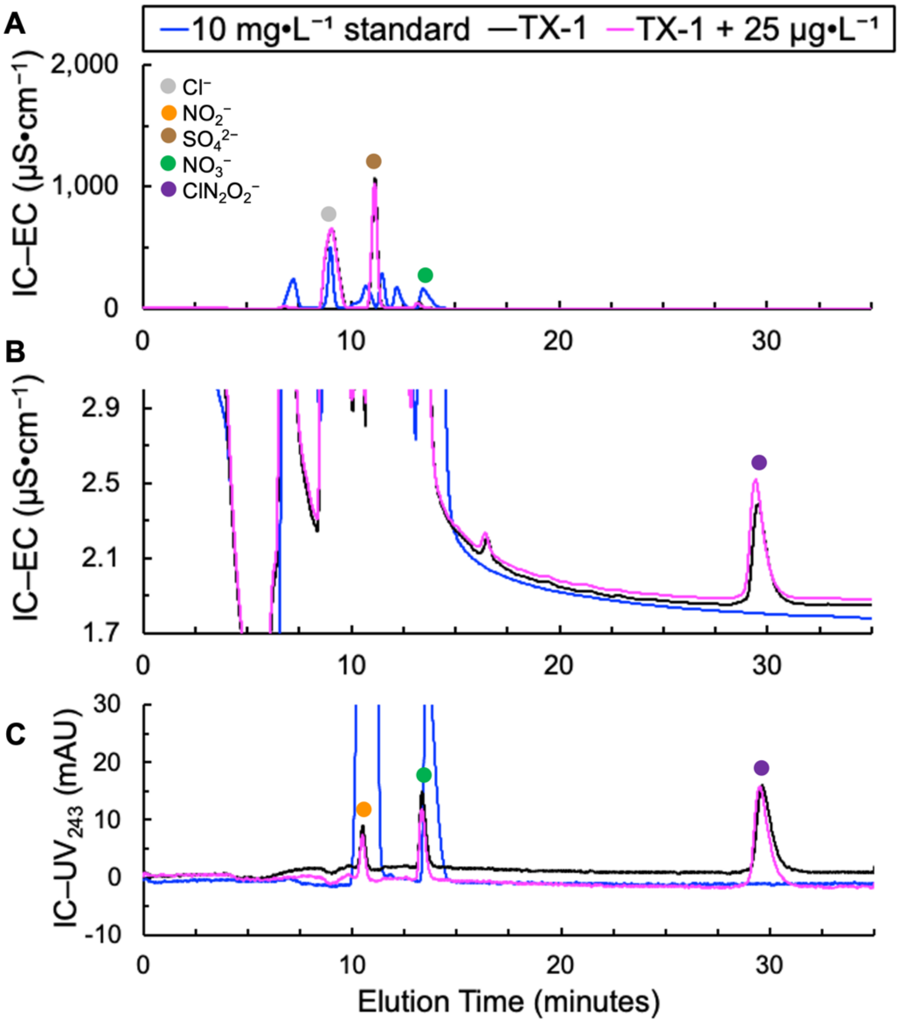

Figure s18. Ion chromatography (IC) chromatograms of a 10 mg•L^{-1} common anion standard, the TX-1 tap water sample, and the TX-1 tap water sample spiked with 25 µg•L^{-1} Cl-N-NO_2^-. (**A**) electrical conductivity (IC-EC), (**B**) IC-EC zoomed in to show the Cl-N-NO_2^- peaks, and (**C**) ultraviolet absorbance at 243 nm (IC-UV_{243}). The colored dots above the peaks indicate the anions detected in the tap water samples.

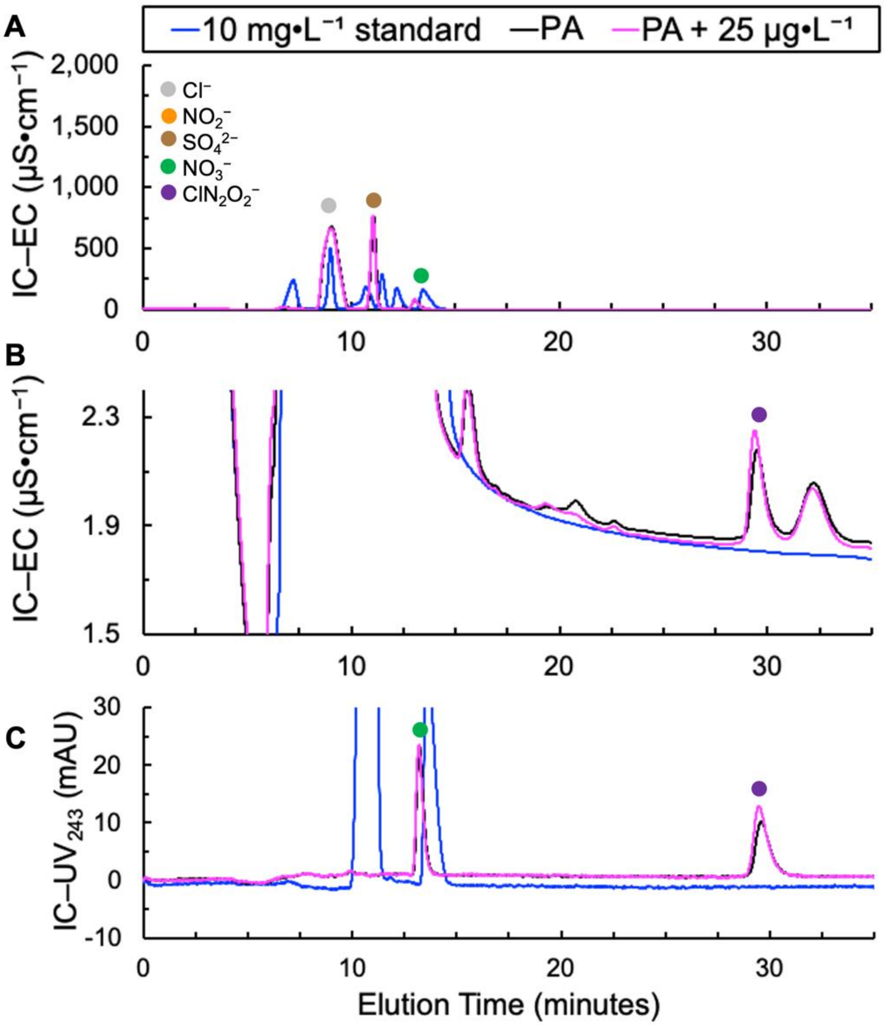

Figure s19. Ion chromatography (IC) chromatograms of a 10 mg•L^{-1} common anion standard, the PA tap water sample, and the PA tap water sample spiked with 25 µg•L^{-1} Cl-N-NO_2^-. (**A**) electrical conductivity (IC-EC), (**B**) IC-EC zoomed in to show the Cl-N-NO_2^- peaks, and (**C**) ultraviolet absorbance at 243 nm (IC-UV_{243}). The colored dots above the peaks indicate the anions detected in the tap water samples.

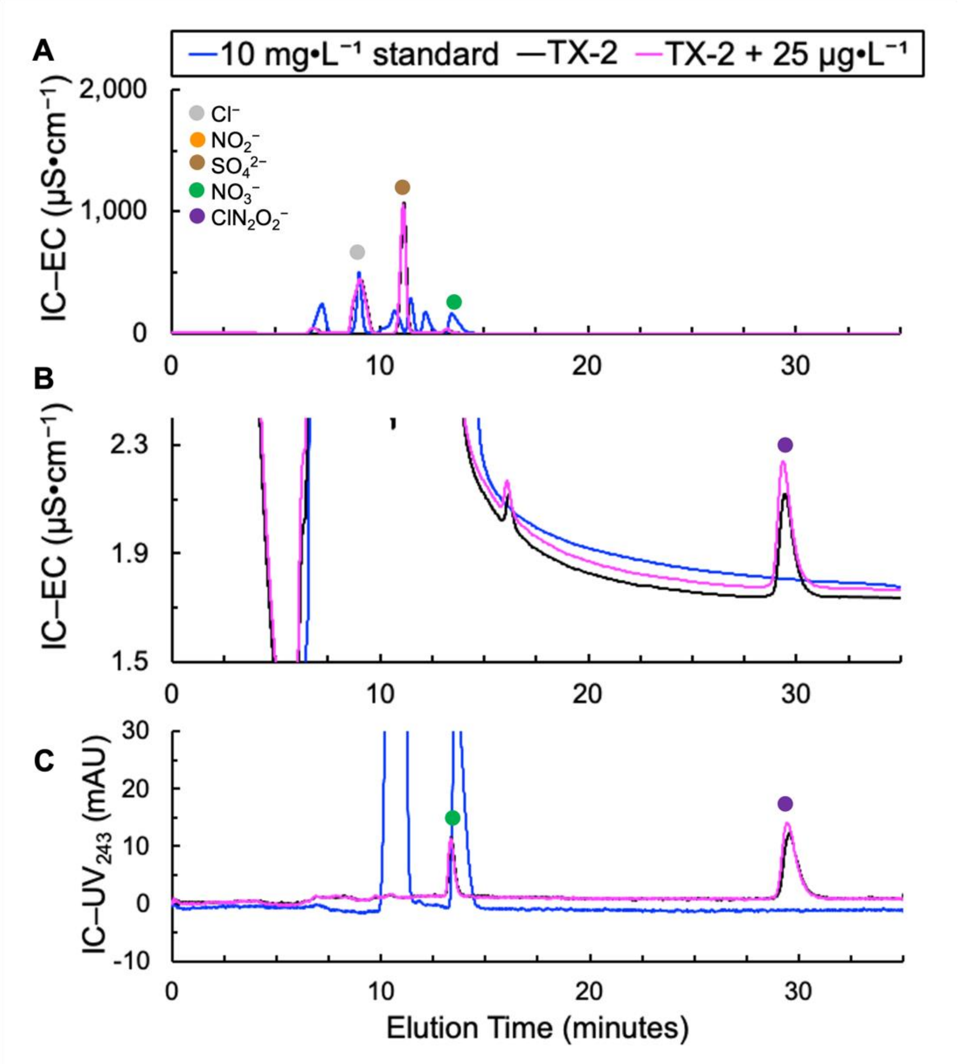

Figure s20. Ion chromatography (IC) chromatograms of a 10 mg•L^{-1} common anion standard, the TX-2 tap water sample, and the TX-2 tap water sample spiked with 25 μg•L^{-1} Cl-N-NO_2^-. (**A**) electrical conductivity (IC-EC), (**B**) IC-EC zoomed in to show the Cl-N-NO_2^- peaks, and (**C**) ultraviolet absorbance at 243 nm (IC-UV_{243}). The colored dots above the peaks indicate the anions detected in the tap water samples.

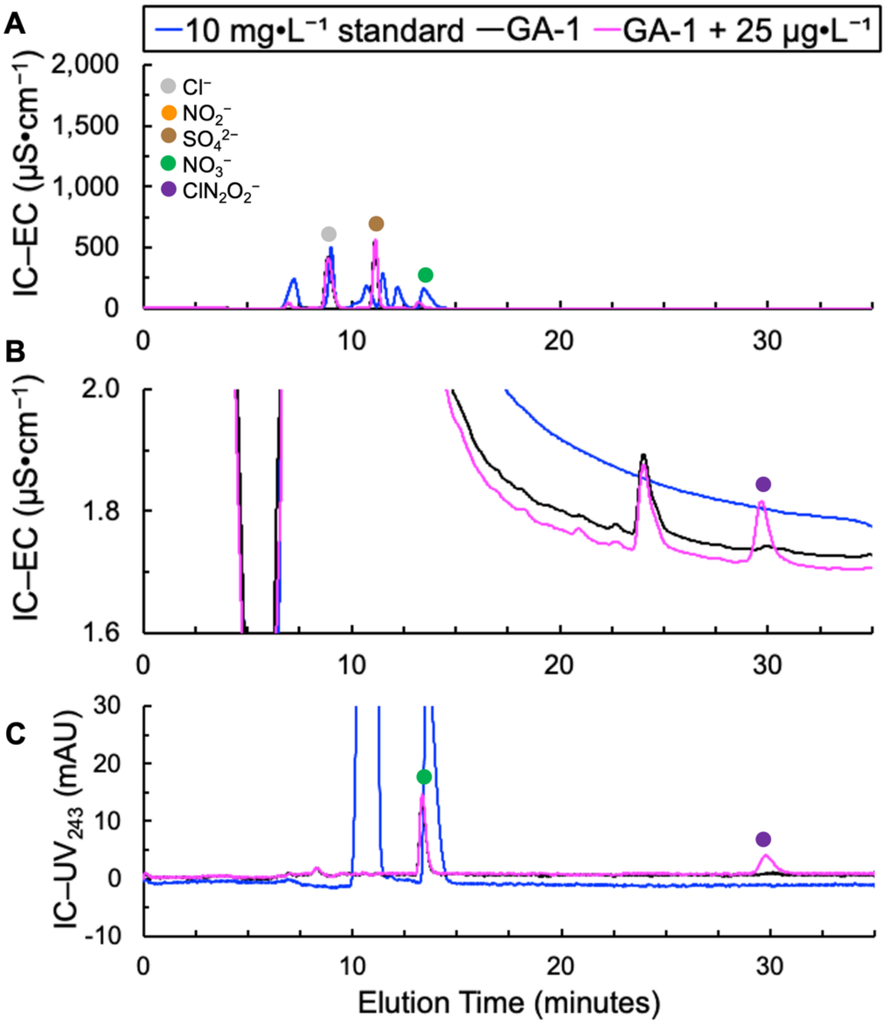

Figure s21. Ion chromatography (IC) chromatograms of a 10 mg•L^{-1} common anion standard, the GA-1 tap water sample, and the GA-1 tap water sample spiked with 25 µg•L^{-1} Cl-N-NO_2^-. (**A**) electrical conductivity (IC-EC), (**B**) IC-EC zoomed in to show the Cl-N-NO_2^- peaks, and (**C**) ultraviolet absorbance at 243 nm (IC-UV_{243}). The colored dots above the peaks indicate the anions detected in the tap water samples.

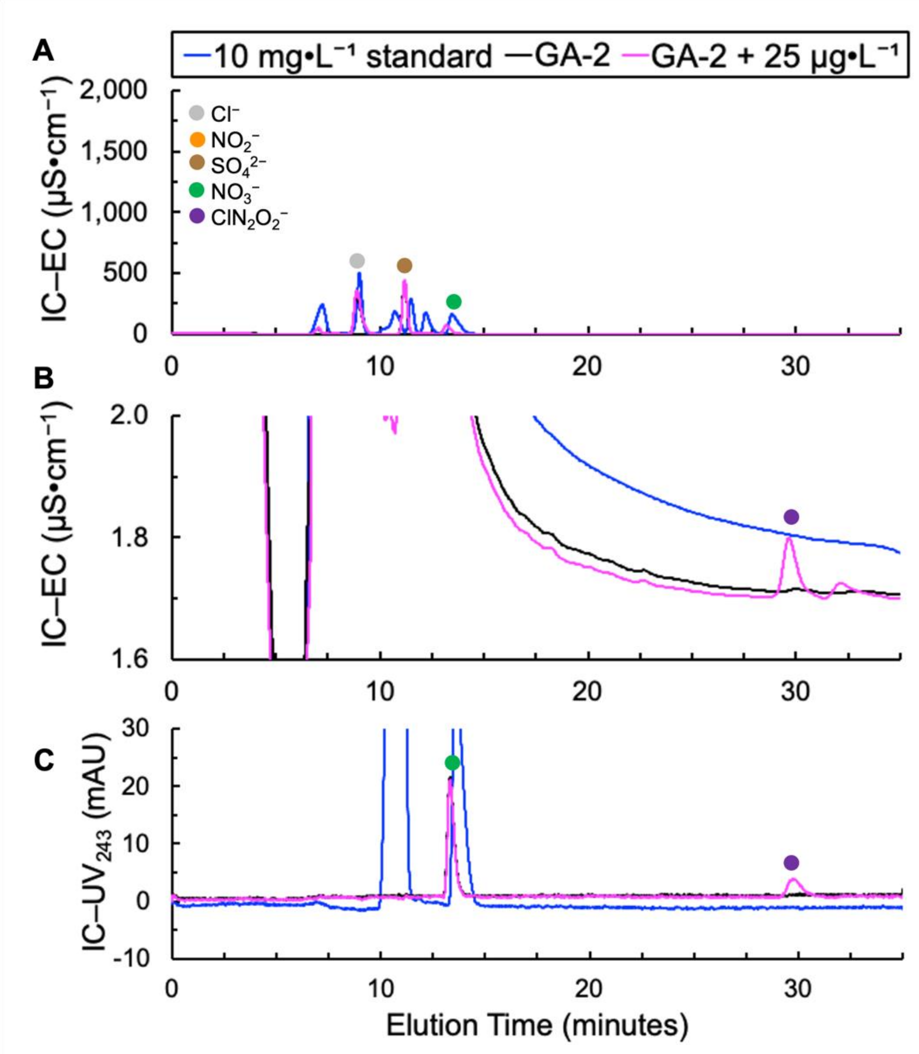

Figure s22. Ion chromatography (IC) chromatograms of a 10 mg•L^{-1} common anion standard, the GA-2 tap water sample, and the GA-2 tap water sample spiked with 25 µg•L^{-1} Cl-N-NO_2^-. (**A**) electrical conductivity (IC-EC), (**B**) IC-EC zoomed in to show the Cl-N-NO_2^- peaks, and (**C**) ultraviolet absorbance at 243 nm (IC-UV_{243}). The colored dots above the peaks indicate the anions detected in the tap water samples.

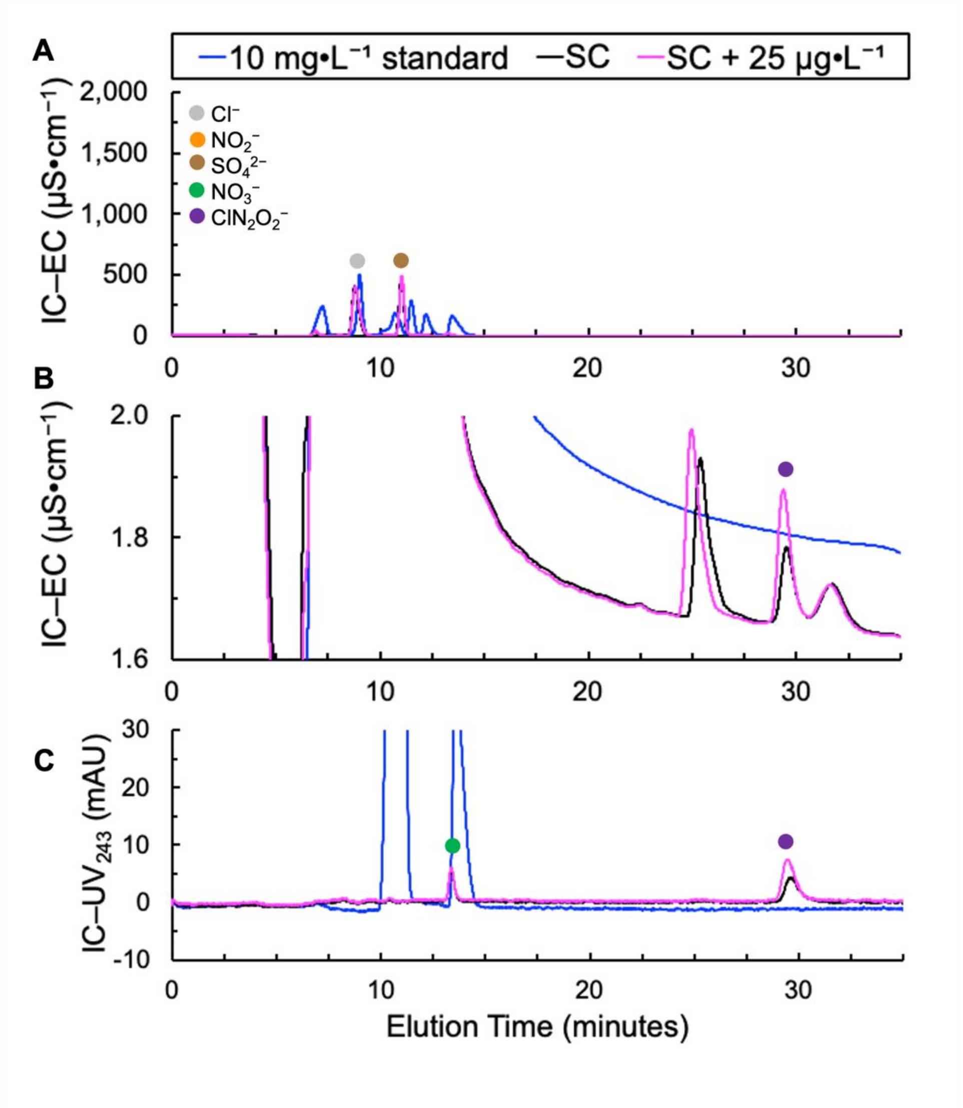

Figure s23. Ion chromatography (IC) chromatograms of a 10 mg•L^{-1} common anion standard, the SC tap water sample, and the SC tap water sample spiked with 25 µg•L^{-1} Cl-N-NO_2^-. (**A**) electrical conductivity (IC-EC), (**B**) IC-EC zoomed in to show the Cl-N-NO_2^- peaks, and (**C**) ultraviolet absorbance at 243 nm (IC-UV_{243}). The colored dots above the peaks indicate the anions detected in the tap water samples.

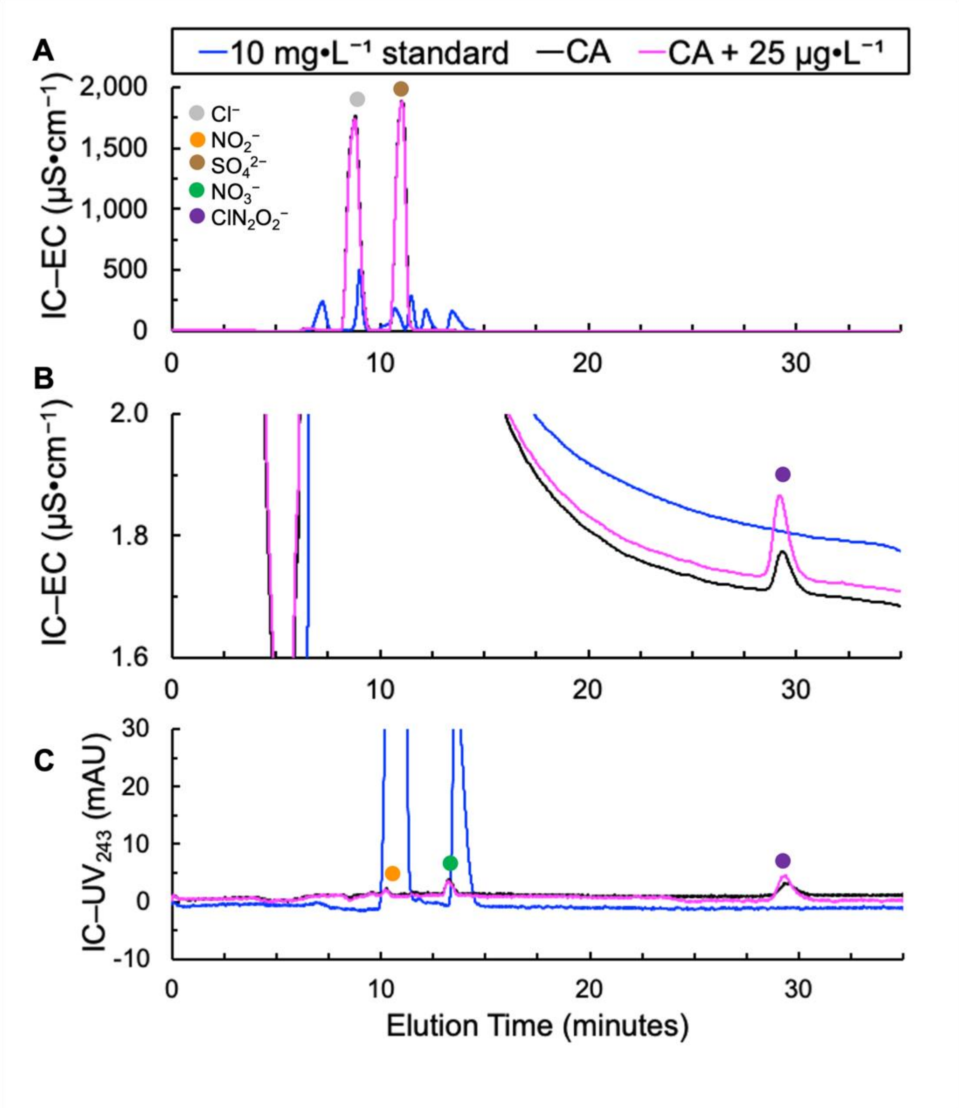

Figure s24. Ion chromatography (IC) chromatograms of a 10 mg•L^{-1} common anion standard, the CA tap water sample, and the CA tap water sample spiked with 25 µg•L^{-1} Cl-N-NO_2^-. (**A**) electrical conductivity (IC-EC), (**B**) IC-EC zoomed in to show the Cl-N-NO_2^- peaks, and (**C**) ultraviolet absorbance at 243 nm (IC-UV_{243}). The colored dots above the peaks indicate the anions detected in the tap water samples.

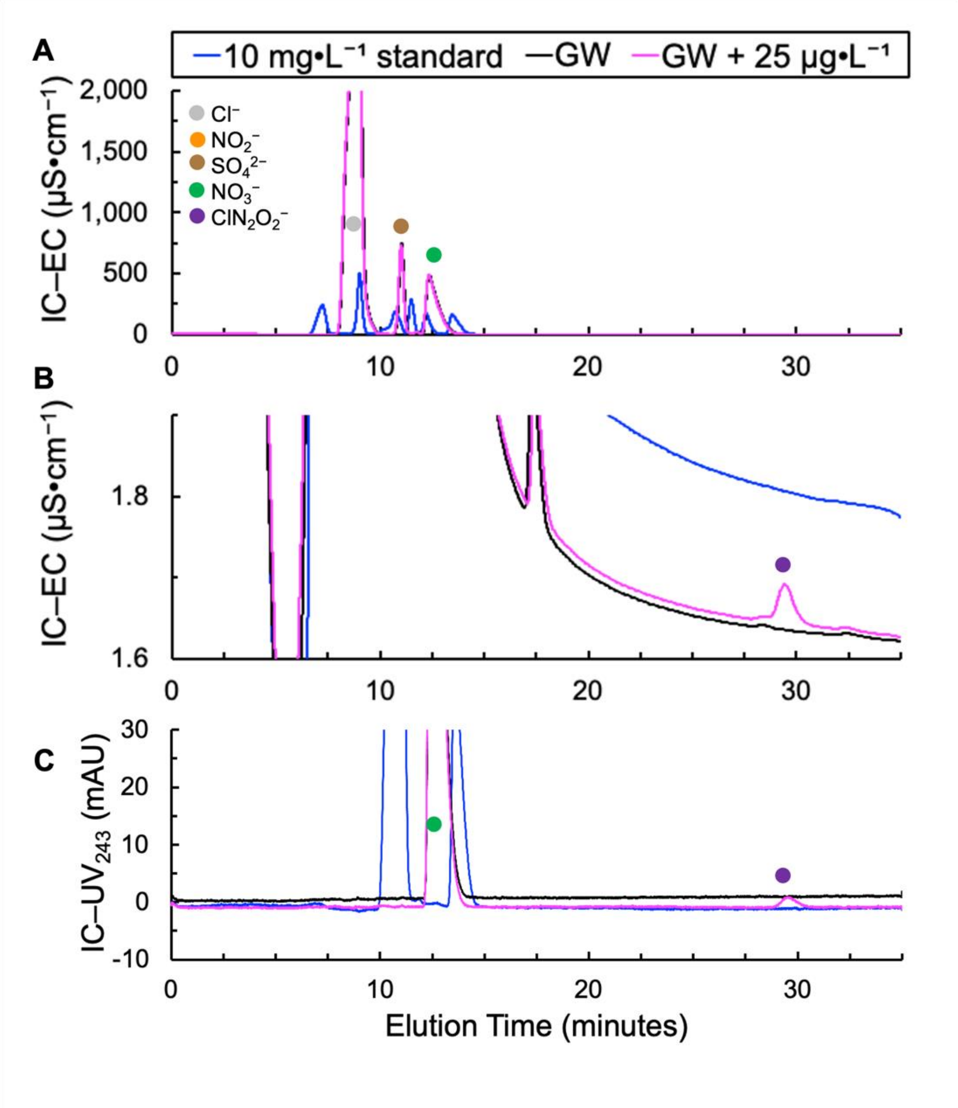

Figure s25. Ion chromatography (IC) chromatograms of a 10 mg•L^{-1} common anion standard, the GW tap water sample, and the GW tap water sample spiked with 25 µg•L^{-1} Cl-N-NO_2^-. (**A**) electrical conductivity (IC-EC), (**B**) IC-EC zoomed in to show the Cl-N-NO_2^- peaks, and (**C**) ultraviolet absorbance at 243 nm (IC-UV_{243}). The colored dots above the peaks indicate the anions detected in the tap water samples.

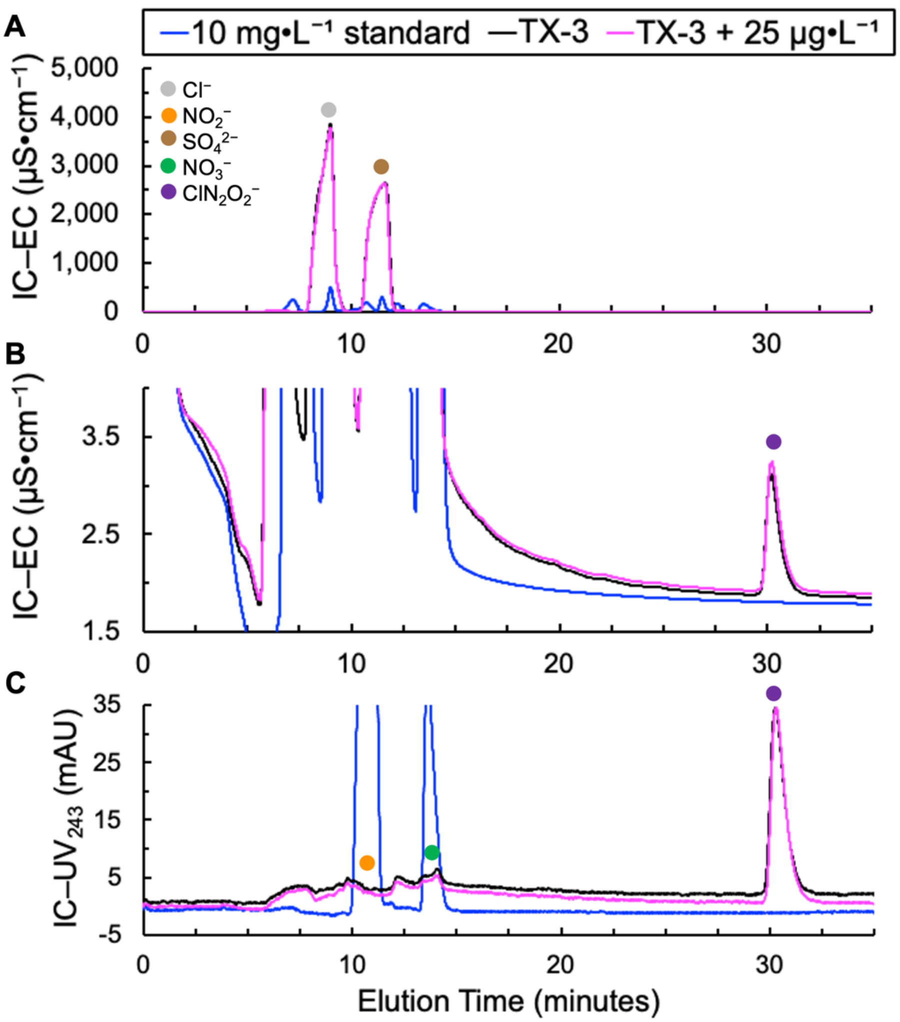

Figure s26. Ion chromatography (IC) chromatograms of a 10 mg•L^{-1} common anion standard, the TX-3 tap water sample, and the TX-3 tap water sample spiked with 25 µg•L^{-1} Cl-N-NO$_2^-$. (**A**) electrical conductivity (IC-EC), (**B**) IC-EC zoomed in to show the Cl-N-NO$_2^-$ peaks, and (**C**) ultraviolet absorbance at 243 nm (IC-UV$_{243}$). The colored dots above the peaks indicate the anions detected in the tap water samples.

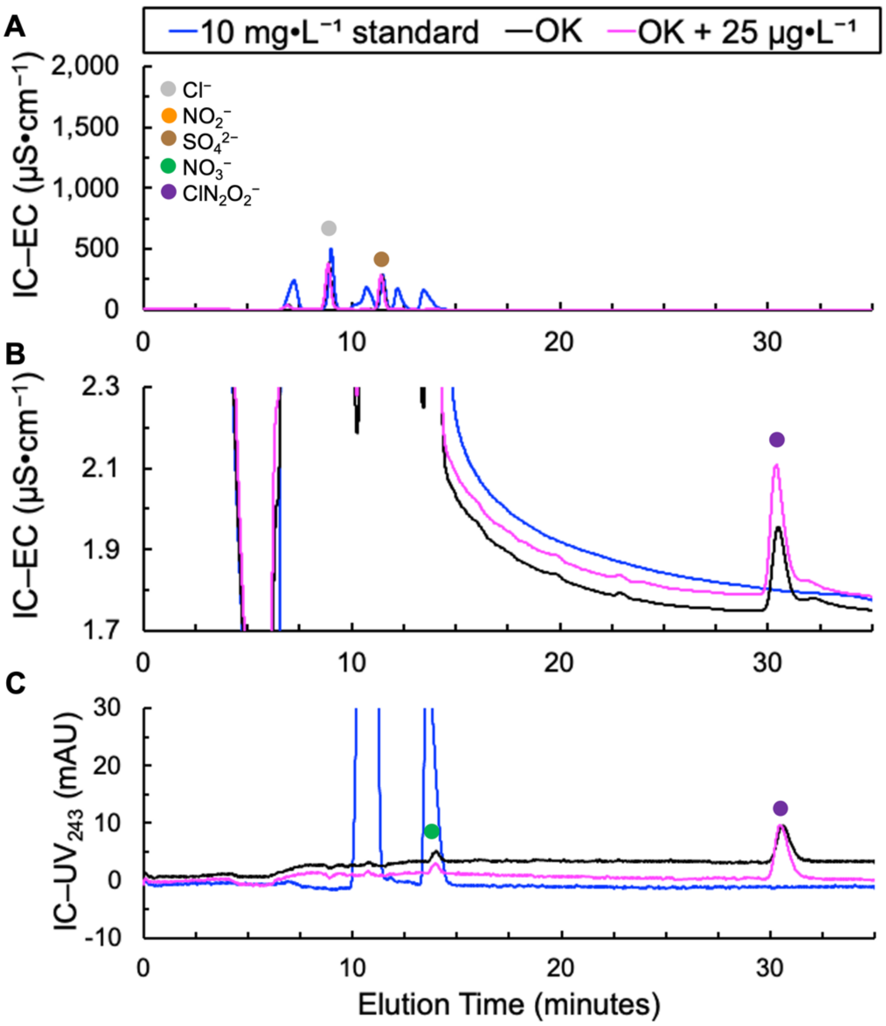

Figure s27. Ion chromatography (IC) chromatograms of a 10 mg•L^{-1} common anion standard, the OK tap water sample, and the OK tap water sample spiked with 25 µg•L^{-1} Cl-N-NO_2^-. (**A**) electrical conductivity (IC-EC), (**B**) IC-EC zoomed in to show the Cl-N-NO_2^- peaks, and (**C**) ultraviolet absorbance at 243 nm (IC-UV_{243}). The colored dots above the peaks indicate the anions detected in the tap water samples.

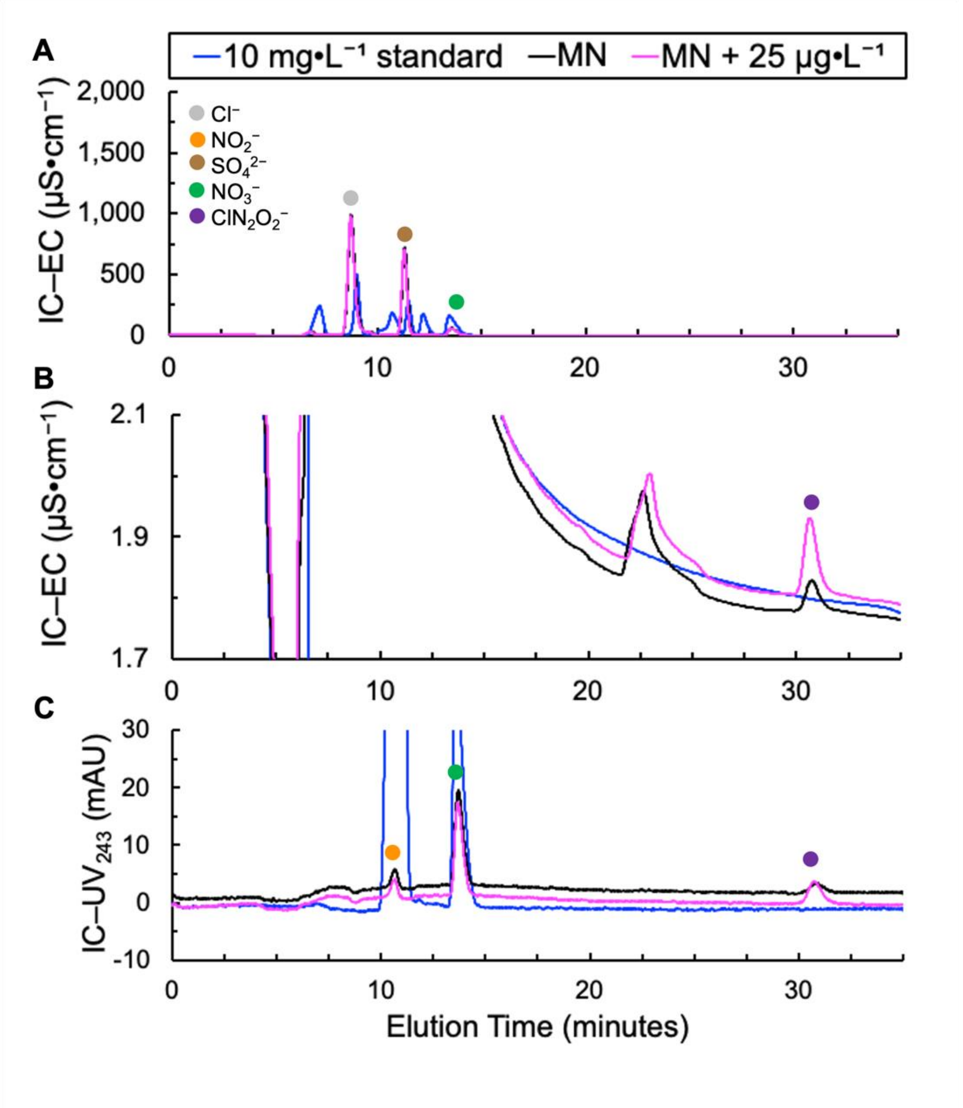

Figure s28. Ion chromatography (IC) chromatograms of a 10 mg•L^{-1} common anion standard, the MN tap water sample, and the MN tap water sample spiked with 25 µg•L^{-1} Cl-N-NO_2^-. (**A**) electrical conductivity (IC-EC), (**B**) IC-EC zoomed in to show the Cl-N-NO_2^- peaks, and (**C**) ultraviolet absorbance at 243 nm (IC-UV_{243}). The colored dots above the peaks indicate the anions detected in the tap water samples.

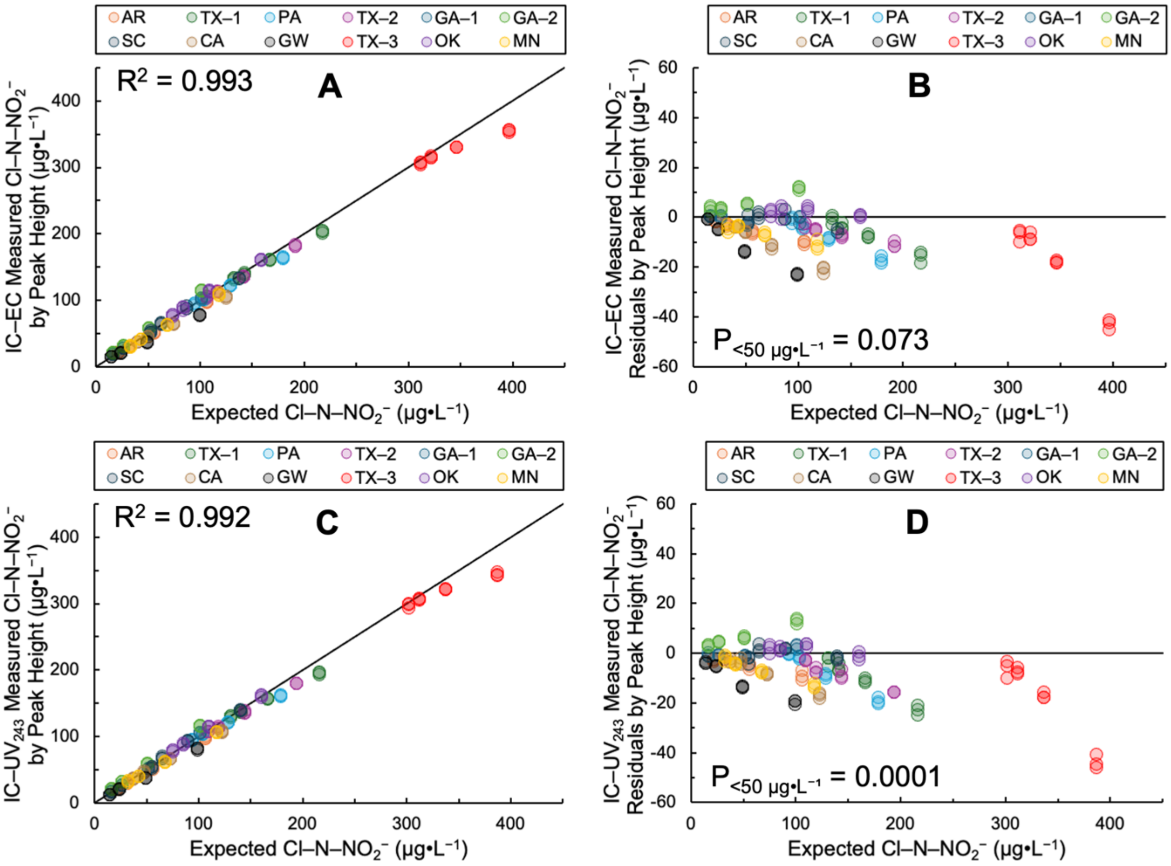

Figure s29. Ion chromatography electrical conductivity (IC-EC, Panels A and B) and ultraviolet absorbance at 243 nm (IC-UV_{243}, Panels C and D) matrix testing with Cl-N-NO_2^- spiked at 15, 25, 50, and 100 µg•L^{-1} into twelve waters which included eight chloraminated tap waters (TX-1, PA, TX-2, SC, CA, TX-3, OK, and MN), three chlorinated tap waters (AR, GA-1, and GA-2), and one synthetic groundwater (GW) with no added disinfectant. (**A**) IC-EC measured by peak height vs. the expected Cl-N-NO_2^-, (**B**) IC-EC residuals by peak height, (**C**) IC-UV_{243} measured by peak height vs. the expected Cl-N-NO_2^-, and (**D**) IC-UV_{243} residuals by peak height. The solid black line in Panels A and C is the 1:1 line and shown with the correlation coefficient, R^2. The P value at the $\alpha = 0.05$ significance level is shown for expected Cl-N-NO_2^- concentrations < 50 µg•L^{-1} ($P_{<50\ \mu g \cdot L^{-1}}$) with a threshold value > 0.05 indicating the residuals have a median indis-tinguishable from zero by the sign test[8] and are therefore unbiased in that concentration range.

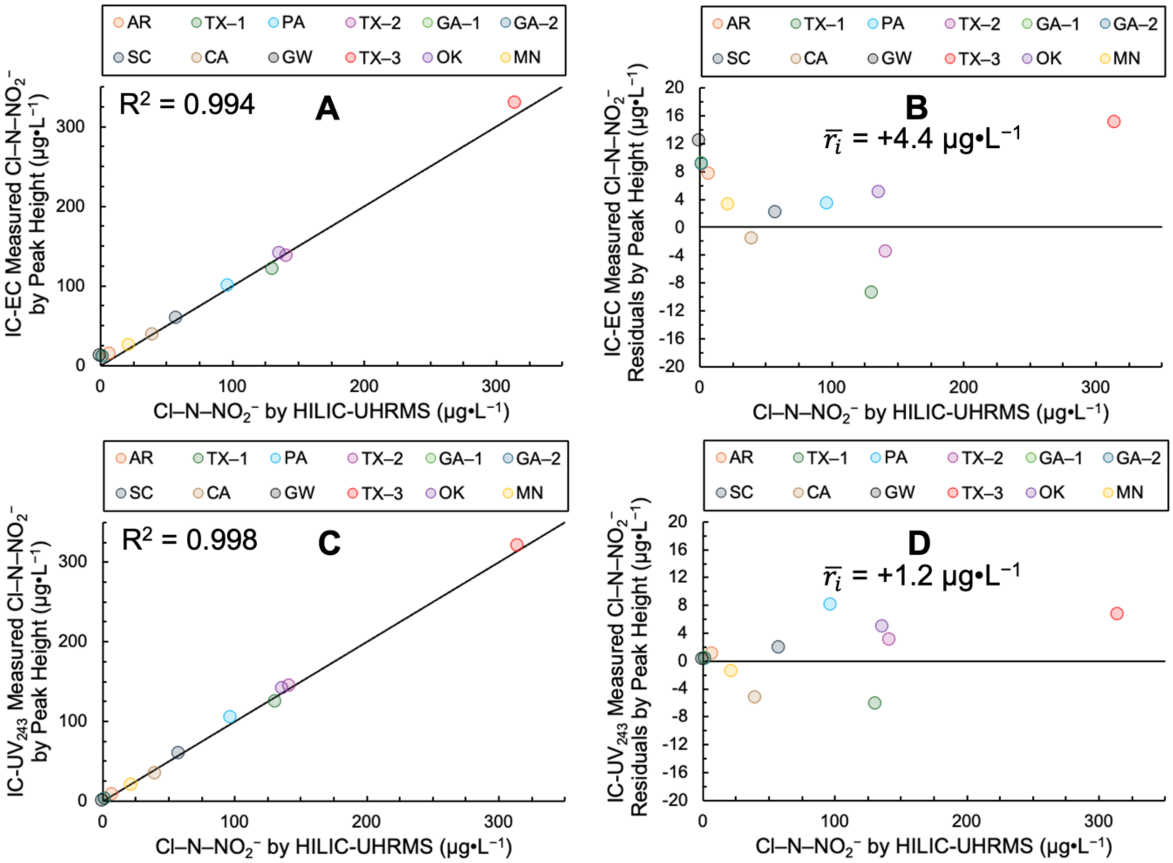

Figure s30. Ion chromatography electrical conductivity (IC-EC, Panels A and B) and ultraviolet absorbance at 243 nm (IC-UV$_{243}$, Panels C and D) comparison testing with HILIC–UHRMS for Cl-N-NO_2^- quantitation in twelve waters which included eight chloraminated tap waters (TX-1, PA, TX-2, SC, CA, TX-3, OK, and MN), three chlorinated tap waters (AR, GA-1, and GA-2), and one synthetic groundwater (GW) with no added disinfectant. (**A**) IC-EC measured by peak height vs. Cl-N-NO_2^- determined by HILIC–UHRMS, (**B**) IC-EC residuals by peak height, (**C**) IC-UV$_{243}$ measured by peak height vs. Cl-N-NO_2^- determined by HILIC–UHRMS, and (**D**) IC-UV$_{243}$ residuals by peak height. The solid black line in Panels A and C is the 1:1 line and shown with the correlation coefficient, R^2. The average residual ($\overline{r_i}$; n = 12) in µg•L^{-1} is shown in Panels B and D as a measure of the linear regression model bias and accuracy with values closer to zero indicating lesser overall bias.

References Cited in the Supporting Information

(1) Fairey, J. L.; Laszakovits, J. R.; Pham, H. T.; Do, T. D.; Hodges, S. D.; McNeill, K.; Wahman, D. G. Chloronitramide anion is a decomposition product of inorganic chloramines. *Science* **2024**, *386* (6724), 882-887. DOI: doi:10.1126/science.adk6749.

(2) USEPA. Definition and Procedure for the Determination of the Method Detection Limit, Revision 2. U.S. Environmental Protection Agency: Washington, D.C., 2016; Vol. EPA 821-R-16-006, https://www.epa.gov/sites/default/files/2016-12/documents/mdl-procedure_rev2_12-13-2016.pdf (accessed 2025-04-25).

(3) Harris, D. C. *Quantitative Chemical Analysis*; W. H. Freeman and Company, **2007**.

(4) Little, T. A. Method Validation Essentials, Limit of Blank, Limit of Detection, and Limit of Quantitation. *Biopharm International* **2015**, *28* (4), 48-51.

(5) Standard Guide for Development and Optimization of D19 Chemical Analysis Methods Intended for EPA Compliance Reporting. ASTM International: 2020, https://compass.astm.org/content-access?contentCode=ASTM%7CD8272-19%7Cen-US (accessed 2025-05-03).

(6) Hach Company. Chlorine, Free (Method 8021) and Total (Method 8167), Low Range. USEPA DPD Method, 0.02 to 2.00 mg/L Cl_2 (LR). Loveland, CO, 2022, https://cdn.hach.com/7FYZVWYB/at/bjxnnjq6pzjbmpns2cvxbf/DOC3165301450_5ed.pdf (accessed 2025-03-15).

(7) Hach Company. Method 10171, Chloramine (Mono). Indophenol Method, 0.04 to 4.50 mg Cl_2/L (LR). Hach Company, Loveland CO: 2015, https://cdn.hach.com/7FYZVWYB/at/5vsg58sffjqgtcfj9c7m5sg/DR_4000_Monochloramine_LR_Method_10171.pdf (accessed 2025-03-15).

(8) Montgomery, D. C., & Runger, G. C. *Applied statistics and probability for engineers*; John Wiley & Sons, **2013**.
